# A Mammalian Mitophagy Receptor, Bcl2-L-13, Recruits the ULK1 Complex to Induce Mitophagy

**DOI:** 10.1016/j.celrep.2018.12.050

**Published:** 2019-01-08

**Authors:** Tomokazu Murakawa, Koji Okamoto, Shigemiki Omiya, Manabu Taneike, Osamu Yamaguchi, Kinya Otsu

**Affiliations:** 1 The School of Cardiovascular Medicine and Sciences, King’s College London British Heart Foundation Centre of Excellence, London SE5 9NU, UK; 2 Laboratory of Mitochondrial Dynamics, Graduate School of Frontier Biosciences, Osaka University, Suita, Osaka 565-0871, Japan; 3 Department of Cardiovascular Medicine, Osaka University Graduate School of Medicine, Suita, Osaka 565-0871, Japan

**Keywords:** Atg32, Bcl2-L-13, mitochondria, mitophagy

## Abstract

Degradation of mitochondria by selective autophagy, termed mitophagy, contributes to the control of mitochondrial quality. Bcl2-L-13 is a mammalian homolog of Atg32, which is an essential mitophagy receptor in yeast. However, the molecular machinery involved in Bcl2-L-13-mediated mitophagy remains to be elucidated. Here, we show that the ULK1 (unc-51-like kinase) complex is required for Bcl2-L-13 to process mitophagy. Screening of a series of yeast Atg mutants revealed that a different set of *ATG* genes is used for Bcl2-L-13- and Atg32-mediated mitophagy in yeast. The components of the Atg1 complex essential for starvation-induced autophagy were indispensable in Bcl2-L-13-, but not Atg32-mediated, mitophagy. The ULK1 complex, a counterpart of the Atg1 complex, is necessary for Bcl2-L-13-mediated mitophagy in mammalian cells. We propose a model where, upon mitophagy induction, Bcl2-L-13 recruits the ULK1 complex to process mitophagy and the interaction of LC3B with ULK1, as well as Bcl2-L-13, is important for the mitophagy.

## Highlights

• Atg32 and Bcl2-L-13 are yeast and mammalian mitophagy receptors, respectively
• Atg1 complex is essential for Bcl2-L-13- but not Atg32-mediated yeast mitophagy
• ULK1 complex is necessary for Bcl2-L-13-mediated mitophagy in mammalian cells

## Graphical Abstract

**Figure:**
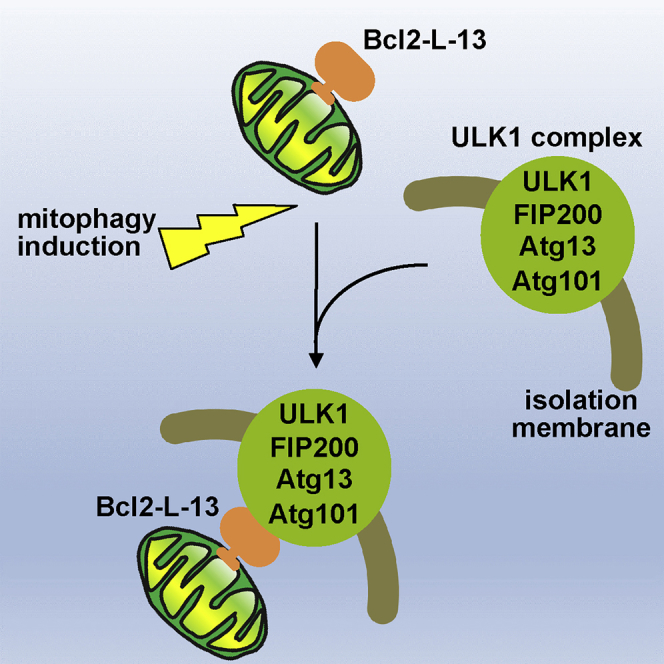

## Teaser

Upon starvation, autophagy degrades cellular components to obtain nutrition for survival. Damaged mitochondria are removed by mitophagy, which is a specific form of autophagy. Bcl2-L-13 protein is involved in the process. Murakawa et al. find that the ULK1 complex, essential for autophagy, is necessary for Bcl2-L-13-mediated mitophagy.

## Introduction

Autophagy is a nonselective degradation system for proteins and organelles induced upon starvation and conserved from yeast to mammals (Tanida, 2011). Recent reports suggest that autophagy can be highly specific. Selective autophagy refers to the selective degradation of organelles, such as mitochondria (mitophagy), peroxisomes (pexophagy), and bacteria (xenophagy).

By taking advantage of yeast genetics, the genes involved in autophagy (autophagy-related [*ATG*] genes) have been identified, which function as the molecular machinery for autophagy (Tsukada and Ohsumi, 1993). A subset of the Atg proteins is required for all types of autophagy, which are referred to as core Atg proteins. The core Atg proteins are categorized into several functional units, such as the Atg1-Atg13 complex, phosphatidylinositol 3-kinase nucleation complex (Atg14, Atg6, Vps15, Vps34, and Atg38), Atg2-Atg18 complex, Atg9, Atg12 conjugation system (Atg5, Atg7, Atg10, Atg12, and Atg16), and Atg8 conjugation system (Atg3, Atg4, Atg7, and Atg8). Specific Atg proteins modulate the function of the core Atg proteins and process their function, such as starvation-induced autophagy (Atg17-Atg29-Atg31 complex), cytoplasm-to-vacuole targeting (Cvt) pathway (Atg11, Atg19, Atg20, Atg21, Atg23, Atg24, and Atg27), pexophagy (Atg11, Atg25, Atg26, Atg28, and Atg30), and mitophagy (Atg11, Atg32, and Atg33). A selective autophagy receptor binds specifically to a cargo and to recruit autophagic machinery to process autophagic sequestration and degradation of a cargo. In yeast, Atg32, which is localized in the mitochondrial outer membrane, is a mitophagy receptor and interacts with Atg8, a ubiquitin-like protein conjugated to the lipid phosphatidylethanolamine through the WXXI motif (an Atg8-interacting motif [AIM]) (Kanki et al., 2009, Okamoto et al., 2009). It interacts with Atg11, a scaffolding protein, which recruits the core Atg protein assembly. Recently, we identified Bcl2-like protein 13 (Bcl2-L-13) as a mammalian functional homolog of Atg32 and found that it is essential for mitophagy in HEK293 cells (Murakawa et al., 2015).

**Figure 1:**
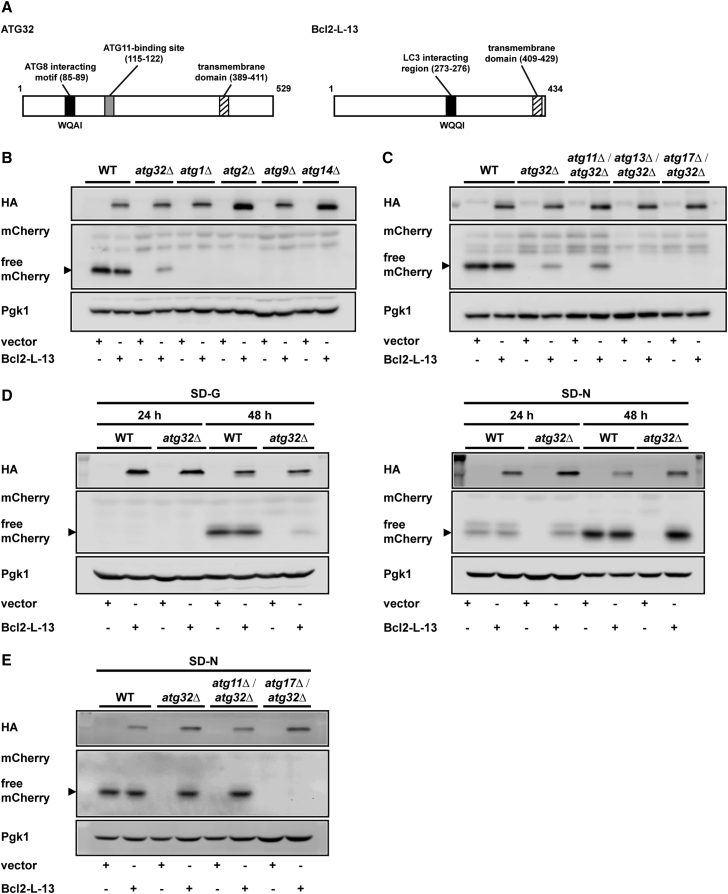
Bcl2-L-13 Requires Atgs Related to Starvation-Specific Autophagy Pathway (A) Schematic representation of ATG32 and mouse Bcl2-L-13 domain structure. (B and C) Yeast cells transfected with HA-Bcl2-L-13 or empty vector were collected 72 hr after induction of mitophagy in glycerol medium (SD-G) and subjected to western blotting for mCherry. Generation of free mCherry is indicated by an arrow-head. Pgk1 was used as a loading control. Yeast strains used are wild-type, *atg32Δ*, *atg1Δ*, *atg2Δ*, *atg9Δ*, *atg14Δ* in (B) and wild-type, *atg32Δ*, *atg11Δ/atg32Δ*, *atg13Δ/atg32Δ, atg17Δ/atg32Δ* in (C). (D) Yeast cells transfected with HA-Bcl2-L-13 or empty vector were collected at indicated time points after the induction of mitophagy by SD-G (left) or nitrogen-starvation medium (SD-N; right). (E) Yeast cells were collected 48 hr after mitophagy induction in SD-N medium. See also Figures S1 and S2.

Bcl2-L-13 shares molecular characteristics, including mitochondrial localization, WXXL or WXXI motifs, acidic amino acid clusters, and single membrane-spanning topology, with Atg32 (Figure 1A) and induces mitochondrial fragmentation and mitophagy in HEK293 cells. Bcl2-L-13 binds to microtubule-associated protein 1A- or 1B-light chain 3B (LC3B), a mammalian homolog of Atg8, through the WXXI motif, an LC3-interacting region (LIR). Bcl2-L-13 exhibits the ability to induce mitophagy in Atg32-deficient yeast, indicating that Bcl2-L-13 is a functional mammalian homolog of Atg32.

In this study, we attempted to identify the molecular machinery for Bcl2-L-13 to induce mitophagy. Our data indicate that the ULK1 complex, which is involved in starvation-induced autophagy, is necessary for Bcl2-L-13-mediated mitophagy in mammalian cells. In addition, LC3B binding to ULK1 as well as Bcl2-L-13 is important for the mitophagy.

## Results and Discussion

### Bcl2-L-13 Requires Atg1, 13, 17, 29, and 31, but Not Atg11, to Induce Mitophagy in Yeast

In this study, it was hypothesized that Bcl2-L-13 uses a similar molecular machinery to induce mitophagy in yeast and in mammalian cells, and a series of mutant yeasts was screened to identify necessary genes for Bcl2-L-13 to induce mitophagy in yeast. In the budding yeast *Saccharomyces cerevisiae*, mitophagy is facilitated during respiratory growth in medium containing glycerol or lactate, a non-fermentable carbon source. A method to monitor mitophagic activity in yeast has been developed using a reporter protein, mitochondrial matrix-targeted dihydrofolate reductase-mCherry protein (mito-dihydrofolate reductase [DHFR]-mCherry) (Kondo-Okamoto et al., 2012). The proteolytic processing of the fusion protein to free mCherry in the vacuole provides a useful marker to assess mitophagy.

First, to investigate which functional unit in the core Atg protein assembly is involved in Bcl2-L-13-mediated mitophagy, the requirement of at least one core Atg protein from each functional unit was tested for Bcl2-L13-mediated mitophagy. We previously reported that Atg7 and Atg8 are involved in Bcl2-L-13-mediated mitophagy in yeast (Murakawa et al., 2015). Thus, the requirement for Bcl2-L13-mediated mitophagy of the remaining four Atg proteins (Atg1, Atg2, Atg9, and Atg14) was examined (Figure 1B; Table 1). Free mCherry was generated in wild-type yeast cells 72 hr after mitophagy induction in glycerol medium (synthetic medium containing 0.5% casamino acids and 0.1% dextrose plus 3% glycerol [SD-G]). The processing of mito-DHFR-mCherry barely occurred in yeast lacking Atg32, and the mitophagy was partially restored in the Atg32-deficient cells expressing Bcl2-L-13 (Murakawa et al., 2015). Expression of Bcl2-L-13 did not lead to the generation of free mCherry in Atg1*-*, Atg2*-*, Atg9*-*, or Atg14-deficient yeast, suggesting that Bcl2-L-13-induced mitophagy is mediated through a known autophagy molecular machinery, including the core Atg proteins.

**Table 1:** *ATG* Gene Requirements for Bcl2-L-13-Mediated Mitophagy in Yeast

Genotype	Mitophagy Restored by Bcl2-L-13	
	Glycerol Medium (SD-G)	Nitrogen Starvation Medium (SD-N)
*atg1Δ*	−	−
*atg2Δ*	−	−
*atg3Δ/atg32Δ*	−	−
*atg4Δ/atg32Δ*	−	−
*atg5Δ/atg32Δ*	−	−
*atg6Δ/atg32Δ*	−	−
*atg7Δ*	−	−
*atg8Δ/atg32Δ*	−	−
*atg9Δ*	−	−
*atg10Δ/atg32Δ*	−	−
*atg11Δ/atg32Δ*	++	++
*atg12Δ/atg32Δ*	−	−
*atg13Δ/atg32Δ*	−	−
*atg14Δ*	−	−
*atg16Δ/atg32Δ*	−	−
*atg17Δ/atg32Δ*	−	−
*atg18Δ/atg32Δ*	−	−
*atg19Δ/atg32Δ*	++	++
*atg20Δ/atg32Δ*	+	+
*atg21Δ/atg32Δ*	−	+
*atg23Δ/atg32Δ*	−	+
*atg24Δ/atg32Δ*	+	+
*atg27Δ/atg32Δ*	+	++
*atg29Δ/atg32Δ*	−	−
*atg31Δ/atg32Δ*	−	−
*atg32Δ*	++	++
*atg33Δ/atg32Δ*	++	++

Mitophagy was assayed by protein degradation assay using mitochondria-targeted mCherry-DHFR-expressing cells grown in glycerol medium for 72 hr or nitrogen starvation medium for 48 hr. Phenotypes are indicated with plus and minus signs as follows: ++, a similar to or higher level than that in *atg32Δ*; +, a significantly higher level than the corresponding empty vector-transfected strain and lower level than *atg32Δ*; −, a similar level to the corresponding vector-transfected strain. See also Figure S1.

For the evaluation of the requirement of other specific Atg proteins, ablation of an *ATG* gene in Atg32-deficient yeast was conducted to exclude the contribution of Atg32 in the induction of mitophagy. Analysis of 27 Atg proteins (Table 1; Figures S1A–S1E) indicates that there were differences in the requirement of Atg proteins between Atg32- and Bcl2-L-13-mediated mitophagy (Table S1). In Atg32-mediated mitophagy, Atg11 was indispensable (Okamoto et al., 2009), whereas Atg11 was not required in Bcl2-L-13-mediated mitophagy (Figure 1C). The results suggest that mitophagy machinery, which Atg32 uses in yeast, may not be conserved in mammalian cells.

Although Atg13, Atg17, Atg29, and Atg31 were not required in Atg32-mediated mitophagy (Okamoto et al., 2009), they were indispensable in Bcl2-L-13-mediated mitophagy (Figures 1C and S1D). Under the starvation condition, the pre-autophagosomal structure (PAS) that functions as a scaffold for the autophagosome formation is labeled with Atg1-Atg13 and Atg17-Atg29-Atg31 complexes (Suzuki and Ohsumi, 2010). Therefore, it is reasonable to assume that Bcl2-L-13 does not use the canonical yeast mitophagy pathway but instead uses the starvation-specific autophagy pathway in yeast. Deletion of Atg proteins, such as Atg21 and Atg23, affected Bcl2-L-13-mediated mitophagy (Figure S1C). Atg21 and Atg23 are related to the Cvt pathway and are also necessary for efficient autophagy (Strømhaug et al., 2004, Tucker et al., 2003). However, the involvement of the Cvt pathway in Bcl2-L-13-mediated mitophagy seems to be unlikely because Atg11 and Atg19, which play a pivotal role in Cvt pathway, were dispensable (Yorimitsu and Klionsky, 2005) (Figures 1C and S1B).

### Bcl2-L-13 Can Completely Restore the Function of Atg32 in Nitrogen Starvation Medium

Then, mitophagy was induced in nitrogen starvation medium (synthetic medium containing 2% dextrose without amino acids and ammonium sulfate [SD-N]), which activates the starvation-specific autophagy pathway. In the wild-type yeast strain, processing of mCherry was observed 48 hr after mitophagy induction (Figure 1D). On the other hand, *atg32Δ* cells transfected with empty vector could not produce free mCherry, suggesting that mitophagy intrinsic to yeast was carried out through the Atg32-mediated pathway. In *atg32Δ* cells, the overexpression of Bcl2-L-13 displayed degradation of mitochondria at a level comparable with that seen in wild-type cells in SD-N medium, whereas a lower level of free mCherry generation was observed in SD-G medium 48 hr after mitophagy induction than in wild-type cells. Bcl2-L-13 induced free mCherry generation in *atg11Δ*/*atg32Δ* but not in *atg17Δ*/*atg32Δ* cells in SD-N medium (Figure 1E). These results support the contention that Bcl2-L-13 uses the starvation-specific autophagy pathway to induce mitophagy in yeast. Because of enhanced mitophagy levels in SD-N medium, the requirement of *ATG* genes was re-evaluated in the Bcl2-L-13-mediated mitophagy (Figures S2A–S2G). The requirement of *ATG* genes in SD-N medium is identical to that in SD-G medium except for Atg21 and Atg23 (Table 1). Overexpression of Bcl2-L-13 produced free mCherry in *atg21Δ*/*atg32Δ* or *atg23Δ*/*atg32Δ* cells, although the level was lower than that seen in *atg32Δ* cells. Atg21 and Atg23 are necessary for efficient autophagy but not indispensable for autophagy (Strømhaug et al., 2004, Tucker et al., 2003). In SD-N medium, autophagy is fully activated so that the contribution of Atg21 and Atg23 to Bcl2-L-13-mediated mitophagy might be relatively smaller than in SD-G medium.

### Atg1-Atg8 Interaction through Atg8-Interacting Motif Is Important for Bcl2-L-13-Mediated Mitophagy in Yeast

**Figure 2:**
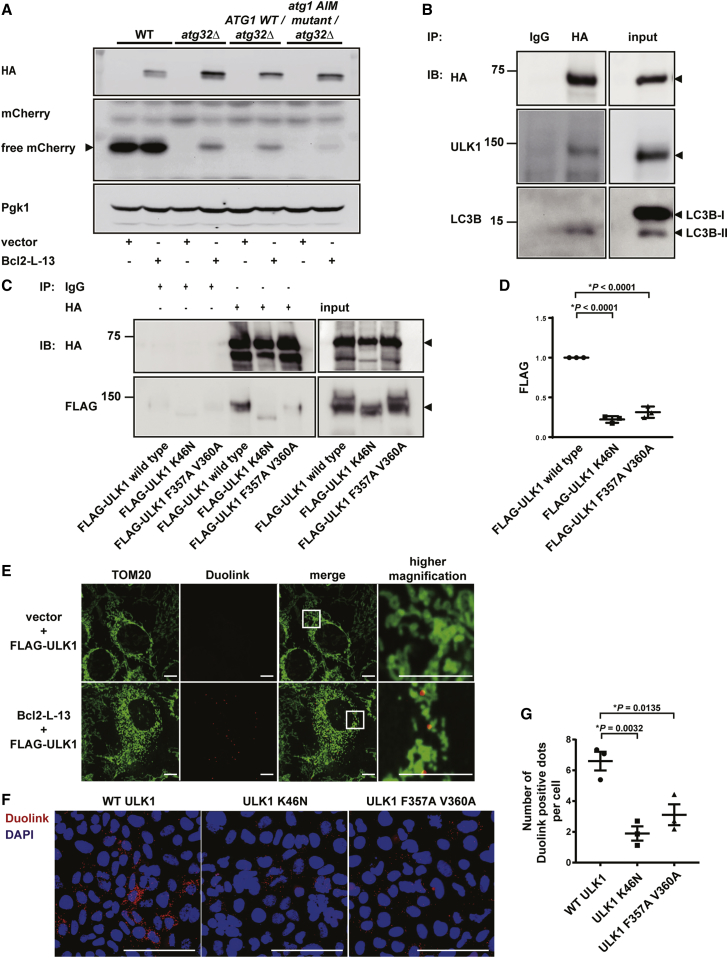
ULK1-LC3B (Atg1-Atg8) Interaction through the LIR (AIM) Motif Is Important for Bcl2-L-13-Mediated Mitophagy (A) The *atg32Δ* yeast cells expressing wild-type Atg1 (*ATG1 WT/atg32Δ*) and AIM mutant of Atg1 (*atg1 AIM mutant/atg32Δ*) transfected with HA-Bcl2-L-13 or empty vector were collected 72 hr after mitophagy induction in SD-G medium. (B) Forty-two hours after transfection with HA-Bcl2-L-13, HEK293A cells were treated with pepstatin A and E64d for 4 hr and immunoprecipitated with anti-HA antibody. Co-precipitated endogenous ULK1 and LC3B were detected by immunoblotting. (C and D) Forty-eight hours after transfection with HA-Bcl2-L-13 and FLAG-tagged wild-type ULK1 or ULK1 mutants, HEK293A cells were immunoprecipitated with anti-HA antibody. Precipitates were immunoblotted with FLAG antibody. Densitometric analysis of the band for FLAG is shown in (D). The value for FLAG-ULK1 wild-type transfected cells in each experiment was set equal to 1 (n = 3). ^∗^p < 0.05. Results are shown as mean ± SEM. (E) Proximity ligation assay. HEK293A cells were transfected with FLAG-ULK1 and empty vector or HA-Bcl2-L-13. After 48 hr of transfection, cells were fixed and stained with Duolink and an anti-TOM20. Scale bar : 10 μm. (F and G) After 48 hr of transfection with HA-Bcl2-L-13 and FLAG tagged wild-type ULK1 or ULK1 mutants, HEK293A cells were stained with Duolink and DAPI. For each experiment, signals from 18 view fields (1674 to 2772 cells) were quantified (n = 3). The number of Duolink positive dots per cell is shown in (G). Scale bar: 100 μm. ^∗^p < 0.05. Results are shown as mean ± SEM. See also Figure S3.

Upon starvation, Atg1 directly interacts with Atg8 in an AIM-dependent manner and recruits Atg8 to autophagosomes (Nakatogawa et al., 2012). To investigate the importance of the Atg1-Atg8 interaction through AIM in Bcl2-L-13-mediated mitophagy, *atg32Δ* cells expressing Atg1 Y429A/V432A, which contains an amino acid substitution in AIM, were generated. GFP-Atg8 was introduced to the strain for the evaluation of autophagic flux (Nakatogawa et al., 2012). Processing of GFP-Atg8 was decreased in Atg1 AIM mutant compared with that in wild-type Atg1 knocked-in yeast upon starvation (Figure S3A). Bcl2-L-13 induced a lower amount of free mCherry in cells expressing Atg1 Y429A/V432A than those expressing wild-type Atg1 (Figure 2A). The Atg1-Atg8 interaction is also required for selective transport of the Atg1-Atg13 complex to the vacuole to control autophagy level and recycle Atg1 and Atg13 during long-time starvation (Kraft et al., 2012). To examine whether this regulatory mechanism affects Bcl2-L-13-mediated mitophagy, the mitophagic activity of Atg1 AIM mutant knocked-in yeast was evaluated 8 hr after mitophagy induction in SD-N medium, where free mCherry was detected (Figure S3B). A lower amount of free mCherry was induced in the Atg1 AIM mutant than that in wild-type Atg1 knocked-in yeast. These results indicate that the Atg1-Atg8 interaction through AIM is important for Bcl2-L-13-mediated mitophagy in yeast.

### Bcl2-L-13 Forms a Complex with ULK1 and LC3B in Mammalian Cells

The Atg1 complex in budding yeast consists of Atg1, Atg13, Atg17, Atg29, and Atg31, whereas its mammalian counterpart, the UNC-51-like kinase (ULK1) complex, comprises Atg1 homolog (ULK1), ATG13, FIP200, and ATG101 (Hosokawa et al., 2009b, Jung et al., 2009). FIP200 has been proposed to be a functional counterpart of Atg17, despite a rather low sequence homology (Hara et al., 2008). In addition, FIP200 shows weak sequence homology in the N-terminal region with Atg11 (Noda and Fujioka, 2015).

LC3B directly interacts with ULK1 through the LIR motif and targets ULK1 to autophagosomes to promote its maturation (Kraft et al., 2012). Bcl2-L-13 has an LIR motif in its sequence and induces mitophagy through the interaction with LC3B (Murakawa et al., 2015). First, HEK293 cells were transfected with HA-Bcl2-L-13, and cell lysates were immunoprecipitated with an anti-hemagglutinin (HA) antibody. Endogenous ULK1 and LC3B-II (microtubule-associated protein IA- or 1B-light chain 3B-II) were detected in the immunoprecipitates, indicating that Bcl2-L-13 may form a complex with ULK1 and LC3B-II in mammalian cells (Figure 2B). Bcl2-L-13 is involved in the mitochondrial uncoupler carbonyl cyanide *m*-chlorophenylhydrazone (CCCP)-induced mitophagy (Murakawa et al., 2015). Endogenous Bcl2-L-13 interacted with ULK1 in CCCP-treated cells (Figure S3C).

### ULK1-LC3B Interaction through the LIR Motif Is Important for the Interaction between Bcl2-L-13 and ULK1

Next, HEK293A cells were transfected with HA-Bcl2-L-13 and FLAG-ULK1 or ULK1 mutant containing an amino acid substitution in the LIR motif (FLAG-ULK1 F357A/V360A) or the catalytic center of the kinase (FLAG-ULK1 K46N). These mutants showed less interaction with Bcl2-L-13 than wild-type ULK1 in the co-immunoprecipitation experiment (Figures 2C and 2D), suggesting ULK1 interacts with LC3B through the LIR motif in the Bcl2-L-13-ULK1 complex and the kinase activity of ULK1 is important for the interaction. To confirm the interaction between Bcl2-L-13 and ULK1, a proximity ligation assay was carried out. In this assay, oligonucleotides attached to antibodies against the two target proteins guide the formation of circular DNA strands when bound in close proximity. The DNA circles, in turn, serve as templates for amplification, allowing individual interacting pairs protein molecules to be visualized (Söderberg et al., 2006). Positive dots were observed on the edge of mitochondria (Figure 2E), and the interaction was decreased in the LIR mutant or kinase-dead mutant (Figures 2F and 2G). Thus, the ULK1-LC3B interaction through the LIR motif is important for forming a complex of Bcl2-L-13 and ULK1.

### Components of the ULK1 Complex Are Important for Bcl2-L-13-Mediated Mitophagy in Mammalian Cells

**Figure 3:**
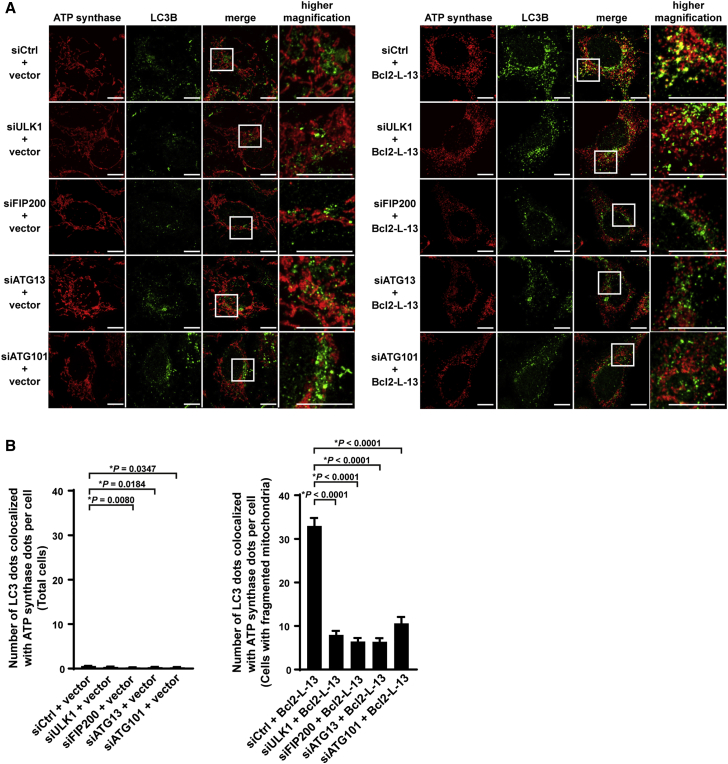
The ULK1 Complex Is Important for Bcl2-L-13-Mediated Mitophagy (A and B) HEK293A cells were transfected by indicated siRNA for 72 hr followed by transfection of empty vector or HA-Bcl2-L-13. Forty-two hours after transfection, cells were treated with 100 nM bafilomycin A1 for 6 hr and immunostained with anti-LC3B and anti-ATP synthase antibodies. Images in the box at higher magnification are shown on the right. Representative fluorescent images are shown in (A) and the number of LC3B dots colocalized with ATP synthase dots per cell is shown in (B). At least 20 cells were counted for each group (n = 3). Scale bar: 10 μm. ^∗^p < 0.05. Results are shown as mean ± SEM. See also Figure S4.

Mitophagy was evaluated by counting LC3B and mitochondrial ATP synthase double-positive dots in the presence of bafilomycin A1. Bafilomycin A1 was used to derive a sufficient number of autophagosomes for analysis. Bcl2-L-13 increased the number of ATP synthase- and LC3B-positive dots in control small interfering RNA (siRNA)-treated cells (Figures 3A and 3B). Knock down of the components of the ULK1 complex, such as ULK1, FIP200, ATG13, and ATG101, decreased the number of ATP synthase and LC3B colocalized dots, indicating that all components of the ULK1 complex are indispensable for Bcl2-L-13-mediated mitophagy (Figures 3A, 3B, S4A, and S4B). To confirm the requirement of the components of the ULK1 complex in physiological condition, mitophagy was induced using CCCP. The number of ATP synthase- and LC3B-positive dots was significantly reduced by knock down of each component of the ULK1 complex (Figures S4C and S4D). Furthermore, expression of the LIR or kinase-dead mutant ULK1 after knock down of ULK1 attenuated Bcl2-L-13-driven mitophagy compared with wild-type ULK1 (Figures S3D and S3E). Although the Atg1 complex is not linked to Atg32-dependent autophagosome formation in yeast (Okamoto et al., 2009), the ULK1 complex targets depolarized mitochondria to recruit downstream ATG proteins in PARKIN-mediated mitophagy as well as Bcl2-L-13-mediated mitophagy (Itakura and Mizushima, 2010). However, in contrast to PARK2/PARKIN-mediated mitophagy, ubiquitination of mitochondrial proteins is not involved in Bcl2-L-13-mediated mitophagy (Chen and Dorn, 2013, Murakawa et al., 2015).

In conclusion, the molecular machinery underlying the induction of mitophagy by Atg32 in yeast is not conserved in Bcl2-L-13-mediated mitophagy in mammalian cells. A complex composed of ULK1, ATG13, FIP200, and ATG101 is vital for Bcl2-L-13-mediated mitophagy induction. LC3B is involved in the interaction between Bcl2-L-13 and the ULK1 complex. Our data indicate a model for Bcl2-L-13-mediated mitophagy in which, upon mitophagy induction, Bcl2-L-13 recruits LC3B to the mitochondrial outer membrane followed by or in coincidence with ULK1 complex recruitment and the interaction between LC3B and ULK1 through LIR (Figure S4E). In addition to ULK1, ATG13 and FIP200 interact with Atg8 family proteins, and they act as scaffolds for assembly for the ULK1 complex (Alemu et al., 2012). Atg8 can multimerize in response to phosphatidylethanolamine conjugation (Kaufmann et al., 2014, Nakatogawa et al., 2007). Thus, LC3B might interconnect between ULK1 and Bcl2-L-13 by oligomerization (Kaufmann et al., 2014, Nakatogawa et al., 2007).

## STAR★Methods

### Key Resources Table

**Table:** 

REAGENT or RESOURCE	SOURCE	IDENTIFIER
**Antibodies**		

Rabbit monoclonal anti-HA (clone C29F4)	Cell Signaling Technology	Cat# 3724; RRID: AB_1549585
Rabbit polyclonal anti-LC3B	Cell Signaling Technology	Cat# 2775; RRID: AB_915950
Rabbit monoclonal anti-ULK1 (clone D8H5)	Cell Signaling Technology	Cat# 8054; RRID: AB_11178668
Rabbit monoclonal anti-FIP200 (clone D10D11)	Cell Signaling Technology	Cat# 12466
Rabbit monoclonal anti-Atg101 (clone E1Z4W)	Cell Signaling Technology	Cat# 13492
Rabbit monoclonal anti-Atg13 (clone D4P1K)	Cell Signaling Technology	Cat# 13273
Mouse monoclonal anti-FLAG (clone M2)	Sigma-Aldrich	Cat# F1804; RRID: AB_262044
Rabbit polyclonal anti-EGFP	Biorbyt	Cat# orb195989
Mouse monoclonal anti-PGK1 (clone 22C5D8)	Abcam	Cat# ab113687; RRID: AB_10861977
Mouse monoclonal anti-mCherry (clone 1C51)	Abcam	Cat# ab125096; RRID: AB_11133266
Rabbit monoclonal anti-TOMM20 (clone EPR15581-54)	Abcam	Cat# ab209606
Mouse monoclonal anti-ATP synthase subunit b (clone 3D5)	Thermo Fisher	Cat# A-21351; RRID: AB_221512
Sheep anti-mouse IgG	GE Healthcare	Cat# NA931; RRID: AB_772210
Donkey anti-rabbit IgG	GE Healthcare	Cat# NA934; RRID: AB_772206

**Chemicals, Peptides, and Recombinant Proteins**		

Penicillin-Streptomycin-Glutamine	GIBCO	Cat# 10378016
geneticin	GIBCO	Cat# 10131-027; CAS: 108321-42-2
nourseothricin	Jena Bioscience	Cat# AB-101S; CAS: 96736-11-7
ScreenFect A	Wako	Cat# 299-73203
RNAi MAX	Invitrogen	Cat# 13778030
Pepstatin A	Sigma	Cat# P5318; CAS: 26305-03-3
E-64d	Sigma	Cat# E8640; CAS: 88321-09-9
Bafilomycin A1	LC Laboratories	Cat# B-1080; CAS: 88899-55-2
ProLong Gold Antifade Mountant with DAPI	Thermo Fisher Scientific	Cat# P36935
Rabbit Immunoglobulin G	Santa Cruz biochemistry	Cat# sc-2027
Protease Inhibitor Cocktail	Sigma	Cat# P8340
ECL Prime Western Blotting Detection Reagent	GE Healthcare Life Science	Cat# RPN2232
Lumigen ECL Ultra	Lumigen	Cat# TMA-100

**Critical Commercial Assays**		

Duolink Detaction Reagent kit	Sigma	Cat# DUO92008
Duolink PLA Rabbit PLUS	Sigma	Cat# DUO92002
Duolink PLA Mouse MINUS	Sigma	Cat# DUO92004
Dynabeads Protein A	Invitrogen	Cat# 1001D

**Experimental Models: Cell Lines**		

Human: HEK293A Cell line	Invitrogen	Cat# R70507; RRID: CVCL_6910

**Experimental Models: Organisms/Strains**		

See Table S2 for yeast strains		N/A

**Oligonucleotides**		

siRNA: ULK1	GE Healthcare Dharmacon	Cat# J-005049-06
siRNA: RB1CC1	GE Healthcare Dharmacon	Cat# J-021117-08
siRNA: ATG101	GE Healthcare Dharmacon	Cat# J-017816-09
siRNA: ATG13	GE Healthcare Dharmacon	Cat# J-020765-12
See Table S3 for primer sequences		N/A

**Recombinant DNA**		

pRS316-GFP-Atg8	Okamoto et al., 2009	N/A
HA-Bcl2-L-13-pcDNA3.1	Murakawa et al., 2015	N/A
p416GPD-Bcl2-L-13(1–407)-TAmito	Murakawa et al., 2015	N/A
p3xFLAG-CMV14-mULK1	Hosokawa et al., 2009a	Addgene; Cat# 24301
p3xFLAG-CMV14-mULK1 K46N	This paper	N/A
p3xFLAG-CMV14-mULK1 F357A V360A	This paper	N/A
pBS-ATG1-kan	Nakatogawa et al., 2012	N/A
pBS-ATG1^AIM mut^-kan	Nakatogawa et al., 2012	N/A
pFA6a-KanMX6	Janke et al., 2004	N/A
pFA6a-natNT2	Janke et al., 2004	N/A

**Software and Algorithms**		

ImageJ (Version 1.51k)	National Institue of Health, USA	https://imagej.nih.gov/ij/index.html
QuPath (Version 0.1.2)	Centre for Cancer Research & Cell Biology, Queen’s University Belfast	https://qupath.github.io/
BZ-X analyzer	Keyence	N/A
GraphPad Prism 7	GraphPad Software	N/A

### Contact for Reagent and Resorce Sharing

Further information and requests for resources and reagents should be directed to and will be fulfilled by the Lead Contact, Kinya Otsu (kinya.otsu@kcl.ac.uk).

### Experimental Model and Subject Details

#### Yeast Strains

The yeast strains used in this study are listed in Table S2. Background strain is *Saccharomyces cerevisiae* BY4741 (*MATa his3Δ1 leu2Δ0 met15Δ0 ura3Δ0*) (Brachmann et al., 1998). To construct *ATG* gene(s) deleted strains, DNA fragments were amplified by PCR using the plasmids pFA6a-*kanMX6* or pFA6a-*natNT2* (Janke et al., 2004) as a template and appropriate pairs of oligonucleotides as primers (Table S3). The pFA6a-*kanMX6* and pFA6a-*natNT2* contain geneticin- and clonNat (nourseothricin)-resistance genes, respectively. Amplified fragments were introduced into KOY1387 or KOY1422 using lithium acetate/polyethylene glycol with herring testis carrier DNA to allow replacement of the chromosomal *ATG* gene by homologous recombination. One to 2 OD_600_ units of cells were washed once with sterile water and incubated in 100 mM lithium acetate at 30°C for 10 min. After lithium acetate was removed, 60% polyethylene glycol, 1M lithium acetate, herring testis DNA, and 2 μg of amplified fragments were added to cell pellets. Cells were mixed throughout and incubated at 30°C for 30 min followed by incubation at 42°C for 20 min. After culturing cells in YPD medium (1% yeast extract, 2% peptone, 2% dextrose) at 30°C for 4 h, cells were spread on YPD plates containing 250 μg/ml geneticin (10131-027, GIBCO) or 100 μg/ml nourseothricin (AB-101S, Jena Bioscience). To construct strains chromosomally expressing the AIM mutant of Atg1 and the corresponding wild-type strain (YMT71 and YMT73), KOY1387 was transformed with PCR products prepared using pBS-*ATG1*-*kan*, pBS-*ATG1*^*AIM mut*^-*kan*, and ATG1-integ-Fw/Rv as primers. The pBS-*ATG1*-*kan* and pBS-*ATG1*^*AIM mut*^-*kan* were gifted by Dr. Nakatogawa (Nakatogawa et al., 2012). They contain wild-type *ATG1* or *ATG1*^*Y429A/V432A*^ together with the *TEF* terminator and the *TEF* promoter followed by the open reading frame of the *kan* gene.

#### Cell Culture

HEK293A cells (RRID: CVCL_6910) were obtained from Invitrogen and were grown in Dulbecco’s modified Eagle’s medium (Sigma) supplemented with 10% fetal bovine serum, and 1% Penicillin-Streptomycin-Glutamine (10378016, GIBCO) at 37°C under 5% CO_2_. Sex of the cells is female.

### Method Details

#### Antibodies

The following primary antibodies were used: anti-HA (#3724), anti-LC3B (#2775), anti-ULK1 (#8054), anti-FIP200 (#12436), anti-ATG101 (#13492), and anti-ATG13 (#13273) from Cell Signaling Technology, anti-FLAG (F1804, Sigma-Aldrich), anti-GFP (orb195989, Biorbyt), anti-Pgk1 (ab113687), anti-mCherry (ab125096), and anti-TOMM20 (ab209606) from abcam, anti-ATP synthase (A-21351, Life Technologies). Secondary antibodies used were the following: Amersham ECL Mouse IgG (NA931, GE Healthcare) and Amersham ECL Rabbit IgG (NA934, GE Healthcare).

#### Transfection

To examine the role of the ULK1 complex in Bcl2-L-13-mediated mitophagy in mammalian cells such as HEK293A cells, the components of the ULK1 complex were knockdowned using their siRNA. Transient transfections were performed using ScreenFect A (299-73203, Wako). After 48 h of transfections, cells were subjected to analysis, unless otherwise indicated. For siRNA-mediated knockdown, cells were transfected with 20 nM ULK1 siRNA (J-005049-06, GE Healthcare Dharmacon), 40 nM RB1CC1 siRNA (J-021117-08, GE Healthcare Dharmacon), 20 nM ATG101 siRNA (J-017816-09, GE Healthcare Dharmacon), and 20 nM ATG13 siRNA (J-020765-12, GE Healthcare Dharmacon) using 3.75 μl/ml of RNAi Max (Invitrogen). Nontargeting siRNA control was obtained from GE Healthcare Dharmacon. After 72 h of transfection, cells were subjected to analysis, unless otherwise indicated.

#### Construction of Plasmids

To generate pRS316-GFP-Atg8, N-terminal GFP-tagged Atg8 under *ATG8* own promoter was inserted into pRS316 (Suzuki et al., 2001). To generate HA-Bcl2-L-13-pcDNA3.1, N-terminal hemagglutinin-tagged mouse Bcl2-L-13 was cloned into pcDNA3.1 (Murakawa et al., 2015). To obtain p416GPD-Bcl2-L-13(1–407)-TAmito, the cytosolic domain of Bcl2-L-13 was fused in frame with a tail-anchor domain (amino acids 618–662) of Gem1, an authentic mitochondrial outer membrane protein in yeast. (Murakawa et al., 2015). The p3xFLAG-CMV14-mULK1 was purchased from Addgene (24301). The p3xFLAG-CMV14-mULK1 K46N and p3xFLAG-CMV14-mULK1 F357A V360A were created by PCR-based site-directed mutagenesis using p3xFLAG-CMV14-mULK1 as a template and pairs of oligonucleotides containing mutations as primers (Table S3).

#### Mitophagy Assay

In this study, it was hypothesized that Bcl2-L-13 utilizes a similar molecular machinery to induce mitophagy in yeast as in mammalian cells and a series of mutant yeasts was screened to identify necessary genes for Bcl2-L-13-mediated mitophagy in yeast. A method using a reporter protein, mitochondrial matrix-targeted dihydrofolate reductase-mCherry protein (mito-dihydrofolate reductase (DHFR)-mCherry) was used to evaluate mitophagic activity in yeast (Kondo-Okamoto et al., 2012). The construct, p416GPD or p416GPD-Bcl2-L-13 (1–407)-TAmito was introduced into yeast strains expressing mitochondrial matrix-targeted DHFR-mCherry using lithium acetate/polyethylene glycol with herring testis carrier DNA.

In the budding yeast *Saccharomyces cerevisiae*, mitophagy is facilitated during respiratory growth in medium containing glycerol or lactate, a non-fermentable carbon source. For mitophagy induction, cells pregrown to mid-log phase (OD_600_ = 1.0-2.0) in SDCA-U (synthetic medium (0.17% yeast nitrogen base without URA) containing 0.5% casamino acids and 2% dextrose) medium were incubated at 30°C for 1–3 days in synthetic medium containing 0.5% casamino acids and 0.1% dextrose plus 3% glycerol (SD-G) or SD-N (0.17% yeast nitrogen base without amino acids and ammonium sulfate, 2% dextrose). For cell lysis, 1 OD_600_ unit of cells were harvested and incubated in 0.1M NaOH at room temperature for 5 min. Then cells were lysed with 50 μL of Laemmli buffer at 100°C for 3 min. After centrifugation, aliquots containing 0.1–0.16 OD_600_ units of cells were subjected to SDS–polyacrylamide gel electrophoresis (SDS-PAGE). Generation of free mCherry was detected by western blot analysis.

#### GFP-Atg8 Processing Assay

A method using a reporter protein, GFP-Atg8, was used to evaluate autophagic activity in yeast. GFP-Atg8 was introduced into *atg32Δ* cells expressing wild-type Atg1 (YMT79) or Atg1 Y429A/V432A (YMT81). GFP-Atg8 processing assay is performed to confirm that the mutation in Atg1 results in attenuated autophagic activity. For induction of starvation-induced autophagy, yeast cells expressing GFP-Atg8 were cultured to mid-log phase (OD_600_ = 1.0-2.0) at 30°C in SDCA-U and then transferred to SD-N. Cells were incubated at 30°C for 6 h. Cell lysates containing 0.16 OD_600_ units of cells were subjected to SDS-PAGE. Generation of free GFP was detected by western blotting.

#### SDS–PAGE and Western Blotting

Cells were washed in ice-cold PBS and lysed in lysis buffer (50 mM Tris-HCl, 137 mM NaCl, 1 mM EDTA, 10% glycerol, 1% Triton X-100, a protease inhibitor cocktail (P8340, Sigma), pH 8.0) on ice. Protein was subjected to SDS–PAGE and transferred to a polyvinylidene difluoride membrane. Membranes were incubated with primary antibodies overnight at 4°C, followed by incubations with secondary antibodies at room temperature (RT) for 1 h. ECL Prime Western Blotting Detection Reagent (RPN2232, GE Healthcare Life Science) or Lumigen ECL Ultra (TMA-100, Lumigen) was used to detect protein.

#### Immunoprecipitation

To investigate if Bcl2-L-13 forms a complex with ULK1 and LC3B and if ULK1–LC3 interaction through the LIR motif is important for the interaction between Bcl2-L-13 and ULK1, an immunoprecipitation experiment was carried out. HEK293A cells were transfected for 48 h and lysed with lysis buffer (50 mM Tris-HCl, 50 mM NaCl, 1 mM EDTA, 0.3% CHAPS, 0.4 mM Na_3_VO_4_, 10 mM NaF, 10 mM sodium pyrophosphate, a protease inhibitor cocktail, pH 7.4). For immunoprecipitation of endogenous ULK1 and LC3B, cells were incubated with 10 μg/ml pepstatin A (P5318, Sigma) and 10 μg/ml E64d (E8640, Sigma) for 4 h before sampling. Lysates were incubated for 10 min at 4°C, followed by centrifugation at 800 *g* for 5 min at 4°C. Then lysates were precleared with 20 μL of magnetic beads-coupled protein A (1001D, Invitrogen) and 1 μg of rabbit immunoglobulin G (sc-2027, Santa Cruz biochemistry). Precleared lysates were subjected to immunoprecipitation using 1 μg of the anti-HA antibody or rabbit immunoglobulin G and 20 μL of magnetic beads-coupled protein A at 4°C for 2 h. The precipitated complexes were washed three times with lysis buffer for immunoblotting.

#### Proximity Ligation Assay

To confirm and characterize the interaction between Bcl2-L-13 and ULK1, a proximity ligation assay was carried out, in which the interaction of molecules can be visualized. HEK293A cells were seeded on a sterile coverslip. The following day, cells were transfected with pcDNA3.1 or HA-Bcl2-L-13-pcDNA3.1 and p3xFLAG-CMV14-mULK1 or its mutants. Cells were fixed with 4% formaldehyde at 37°C for 10 min, permeabilized with 0.2% Triton X-100 for 15 min, and then blocked with Duolink blocking solution for 30 min and incubated overnight at 4°C with anti-HA and anti-Flag antibodies diluted 1:200 in Duolink blocking solution. After washing with Duolink *In Situ* Wash Buffer A (DUO82049, Sigma), cells were incubated with Duolink PLA Rabbit PLUS (DUO92002, Sigma) and PLA Mouse MINUS (DUO92004, Sigma) proximity probes and proximity ligation was performed using the Duolink Detection Reagent kit (DUO92008, Sigma). After washing with Duolink *In Situ* Wash Buffer B, cells were mounted with ProLong Gold Antifade Mountant with DAPI (P36935, Thermo Fisher Scientific). Images were acquired using a Nikon Ti-Eclipse inverted microscope (Nikon) or a BZ-X700 fluorescent microscope (Keyence) with a CFI Plan Apo λ20x objective lens (Nikon) and analyzed with BZ-X Analyzer (Keyence), NIH ImageJ software (version 1.51k), and QuPath (Version 0.1.2). The number of the fluorescent-positive dots from reaction products was counted and standardized by cell number, which was estimated from DAPI staining of the nucleus. Values from empty vector transfected groups were subtracted from corresponding HA-Bcl2-L-13-pcDNA3.1 transfected groups as background.

#### Microscopy for Assessment of Mitophagy

Mitophagy was evaluated by counting LC3B and mitochondrial ATP synthase double positive dots in the presence of bafilomycin A1. Bafilomycin A1 was used to derive a sufficient number of autophagosomes for analysis. HEK293A cells were transfected with siRNA using RNAi Max. Seventy-two h after the siRNA transfection, cells were split and transfected with the plasmids using ScreenFect A. Forty-two h after the plasmid DNA transfection, 100 nM bafilomycin A1 was added for 6 h. Cells were fixed and permeabilized with methanol for 10 min at −20°C. Cells were immunostained with anti-ATP synthase (Life Technologies, 1:200) and anti-LC3B (Cell Signaling Technology, 1:100) antibody overnight at 4°C followed by incubation with secondary antibodies for 1 h at RT. After washing, cells were mounted with ProLong Gold Antifade Mountant and analyzed using a Nikon Ti-Eclipse inverted microscope (Nikon) equipped with a Yokagawa CSU-X1-M2 spinning disk unit (Yokagawa) and an Andor Neo sCMOS camera (Andor Technology). Mitophagy was evaluated by counting the number of ATP synthase dots colocalized with LC3B dots. At least 20 cells were quantified for each group.

### Quantification and Statistics Analysis

The number of independent biological repeats (*n*) is shown in the figure legends and recorded in detail below. No samples were excluded in analysis. No prior estimation of sample sizes or test for normal distribution was conducted. Results are shown as mean ± SEM. Statistical analyses were performed using GraphPad Prism 7 (GraphPad Software). Paired data were evaluated by unpaired, two-tailed t test. A one-way analysis of variance (ANOVA) followed by Tukey–Kramer’s post hoc test was used for multiple comparisons. A value of p < 0.05 was considered statistically significant.

Figure 2D.

For all groups, n = 3. FLAG-ULK1 wild-type versus FLAG-ULK1 K46N p < 0.0001, FLAG-ULK1 wild-type versus FLAG-ULK1 F357A V360A p < 0.0001, FLAG-ULK1 K46N versus FLAG-ULK1 F357A V360A p = 0.1260.

A one-way ANOVA followed by Tukey–Kramer’s post hoc test.

Figure 2G.

For all groups, n = 3. WT ULK1 versus ULK1 K46N p = 0.0032, WT ULK1 versus ULK1 F357A V360A p = 0.0135, ULK1 K46N versus ULK1 F357A V360A p = 0.3730.

A one-way ANOVA followed by Tukey–Kramer’s post hoc test.

Figure 3B.

For all groups, n = 3. siCtrl + vector versus siULK1 + vector p = 0.1975, siCtrl + vector versus siFIP200 + vector p = 0.0080, siCtrl + vector versus siATG13 + vector p = 0.0184, siCtrl + vector versus siATG101 + vector p = 0.0347, siULK1 + vector versus siFIP200 + vector p = 0.2886, siULK1 + vector versus siATG13 + vector p = 0.5526, siULK1 + vector versus siATG101 + vector p = 0.7804, siFIP200 + vector versus siATG13 + vector p = 0.9795, siFIP200 + vector versus siATG101 + vector p = 0.8660, siATG13 + vector versus siATG101 + vector p = 0.9930.

siCtrl + Bcl2-L-13 versus siULK1 + Bcl2-L-13 p < 0.0001, siCtrl + Bcl2-L-13 versus siFIP200 + Bcl2-L-13 p < 0.0001, siCtrl + Bcl2-L-13 versus siATG13 + Bcl2-L-13 p < 0.0001, siCtrl + Bcl2-L-13 versus siATG101 + Bcl2-L-13 p < 0.0001, siULK1 + Bcl2-L-13 versus siFIP200 + Bcl2-L-13 p = 0.9069, siULK1 + Bcl2-L-13 versus siATG13 + Bcl2-L-13 p = 0.8946, siULK1 + Bcl2-L-13 versus siATG101 + Bcl2-L-13 p = 0.5738, siFIP200 + Bcl2-L-13 versus siATG13 + Bcl2-L-13 p > 0.9999, siFIP200 + Bcl2-L-13 versus siATG101 + Bcl2-L-13 p = 0.1996, siATG13 + Bcl2-L-13 versus siATG101 + Bcl2-L-13 p = 0.1901.

A one-way ANOVA followed by Tukey–Kramer’s post hoc test.

Table 1, Figure S1E.

For all groups, n = 3. *atg32Δ* + Bcl2-L-13 versus *atg1Δ* + vector p < 0.0001, *atg32Δ* + Bcl2-L-13 versus *atg1Δ* + Bcl2-L-13 p < 0.0001, *atg1Δ* + vector versus *atg1Δ* + Bcl2-L-13 p = 0.8162, *atg32Δ* + Bcl2-L-13 versus *atg2Δ* + vector p < 0.0001, *atg32Δ* + Bcl2-L-13 versus *atg2Δ* + Bcl2-L-13 p < 0.0001, *atg2Δ* + vector versus *atg2Δ* + Bcl2-L-13 p = 0.9829, *atg32Δ* + Bcl2-L-13 versus *atg3Δ / atg32Δ* + vector p < 0.0001, *atg32Δ* + Bcl2-L-13 versus *atg3Δ / atg32Δ* + Bcl2-L-13 p < 0.0001, *atg3Δ / atg32Δ* + vector versus *atg3Δ / atg32Δ* + Bcl2-L-13 p = 0.8898, *atg32Δ* + Bcl2-L-13 versus *atg4Δ / atg32Δ* + vector p < 0.0001, *atg32Δ* + Bcl2-L-13 versus *atg4Δ / atg32Δ* + Bcl2-L-13 p < 0.0001, *atg4Δ / atg32Δ* + vector versus *atg4Δ / atg32Δ* + Bcl2-L-13 p = 0.7095, *atg32Δ* + Bcl2-L-13 versus *atg5Δ / atg32Δ* + vector p < 0.0001, *atg32Δ* + Bcl2-L-13 versus *atg5Δ / atg32Δ* + Bcl2-L-13 p < 0.0001, *atg5Δ / atg32Δ* + vector versus *atg5Δ / atg32Δ* + Bcl2-L-13 p = 0.5966, *atg32Δ* + Bcl2-L-13 versus *atg6Δ / atg32Δ* + vector p < 0.0001, *atg32Δ* + Bcl2-L-13 versus *atg6Δ / atg32Δ* + Bcl2-L-13 p < 0.0001, *atg6Δ / atg32Δ* + vector versus *atg6Δ / atg32Δ* + Bcl2-L-13 p = 0.7969, *atg32Δ* + Bcl2-L-13 versus *atg8Δ / atg32Δ* + vector p < 0.0001, *atg32Δ* + Bcl2-L-13 versus *atg8Δ / atg32Δ* + Bcl2-L-13 p < 0.0001, *atg8Δ / atg32Δ* + vector versus *atg8Δ / atg32Δ* + Bcl2-L-13 p = 0.8569, *atg32Δ* + Bcl2-L-13 versus *atg9Δ* + vector p < 0.0001, *atg32Δ* + Bcl2-L-13 versus *atg9Δ* + Bcl2-L-13 p < 0.0001, *atg9Δ* + vector versus *atg9Δ* + Bcl2-L-13 p = 0.8162, *atg32Δ* + Bcl2-L-13 versus *atg10Δ / atg32Δ* + vector p < 0.0001, *atg32Δ* + Bcl2-L-13 versus *atg10Δ / atg32Δ* + Bcl2-L-13 p < 0.0001, *atg10Δ / atg32Δ* + vector versus *atg10Δ / atg32Δ* + Bcl2-L-13 p = 0.9997, *atg32Δ* + Bcl2-L-13 versus *atg11Δ / atg32Δ* + vector p = 0.0012, *atg32Δ* + Bcl2-L-13 versus *atg11Δ / atg32Δ* + Bcl2-L-13 p = 0.0031, *atg11Δ / atg32Δ* + vector versus *atg11Δ / atg32Δ* + Bcl2-L-13 p < 0.0001, *atg32Δ* + Bcl2-L-13 versus *atg12Δ / atg32Δ* + vector p < 0.0001, *atg32Δ* + Bcl2-L-13 versus *atg12Δ / atg32Δ* + Bcl2-L-13 p < 0.0001, *atg12Δ / atg32Δ* + vector versus *atg12Δ / atg32Δ* + Bcl2-L-13 p = 0.8646, *atg32Δ* + Bcl2-L-13 versus *atg13Δ / atg32Δ* + vector p < 0.0001, *atg32Δ* + Bcl2-L-13 versus *atg13Δ / atg32Δ* + Bcl2-L-13 p < 0.0001, *atg13Δ / atg32Δ* + vector versus *atg13Δ / atg32Δ* + Bcl2-L-13 p = 0.9646, *atg32Δ* + Bcl2-L-13 versus *atg14Δ* + vector p < 0.0001, *atg32Δ* + Bcl2-L-13 versus *atg14Δ* + Bcl2-L-13 p < 0.0001, *atg14Δ* + vector versus *atg14Δ* + Bcl2-L-13 p = 0.9559, *atg32Δ* + Bcl2-L-13 versus *atg16Δ / atg32Δ* + vector p < 0.0001, *atg32Δ* + Bcl2-L-13 versus *atg16Δ / atg32Δ* + Bcl2-L-13 p < 0.0001, *atg16Δ / atg32Δ* + vector versus *atg16Δ / atg32Δ* + Bcl2-L-13 p = 0.9621, *atg32Δ* + Bcl2-L-13 versus *atg17Δ / atg32Δ* + vector p < 0.0001, *atg32Δ* + Bcl2-L-13 versus *atg17Δ /atg32Δ* + Bcl2-L-13 p < 0.0001, *atg17Δ / atg32Δ* + vector versus *atg17Δ / atg32Δ* + Bcl2-L-13 p = 0.3782, *atg32Δ* + Bcl2-L-13 versus *atg18Δ / atg32Δ /* + vector p < 0.0001, *atg32Δ* + Bcl2-L-13 versus *atg18Δ / atg32Δ* + Bcl2-L-13 p < 0.0001, *atg18Δ / atg32Δ* + vector versus *atg18Δ / atg32Δ* + Bcl2-L-13 p = 0.8983, *atg32Δ* + Bcl2-L-13 versus *atg19Δ / atg32Δ* + vector p = 0.0137, *atg32Δ* + Bcl2-L-13 versus *atg19Δ / atg32Δ* + Bcl2-L-13 p = 0.7498, *atg19Δ / atg32Δ* + vector versus *atg19Δ / atg32Δ* + Bcl2-L-13 p = 0.0063, *atg32Δ* + Bcl2-L-13 versus *atg20Δ / atg32Δ* + vector p < 0.0001, *atg32Δ* + Bcl2-L-13 versus *atg20Δ / atg32Δ* + Bcl2-L-13 p < 0.0001, *atg20Δ / atg32Δ* + vector versus *atg20Δ / atg32Δ* + Bcl2-L-13 p = 0.0055, *atg32Δ* + Bcl2-L-13 versus *atg21Δ / atg32Δ* + vector p < 0.0001, *atg32Δ* + Bcl2-L-13 versus *atg21Δ / atg32Δ* + Bcl2-L-13 p < 0.0001, *atg21Δ / atg32Δ* + vector versus *atg21Δ / atg32Δ* + Bcl2-L-13 p = 0.9904, *atg32Δ* + Bcl2-L-13 versus *atg23Δ / atg32Δ* + vector p < 0.0001, *atg32Δ* + Bcl2-L-13 versus *atg23Δ / atg32Δ* + Bcl2-L-13 p < 0.0001, *atg23Δ / atg32Δ* + vector versus *atg23Δ / atg32Δ* + Bcl2-L-13 p = 0.3941, *atg32Δ* + Bcl2-L-13 versus *atg24Δ / atg32Δ* + vector p < 0.0001, *atg32Δ* + Bcl2-L-13 versus *atg24Δ / atg32Δ* + Bcl2-L-13 p < 0.0001, *atg24Δ / atg32Δ* + vector versus *atg24Δ / atg32Δ* + Bcl2-L-13 p = 0.0004, *atg32Δ* + Bcl2-L-13 versus *atg27Δ / atg32Δ* + vector p = 0.0001, *atg32Δ* + Bcl2-L-13 versus *atg27Δ / atg32Δ* + Bcl2-L-13 p = 0.0039, *atg27Δ / atg32Δ* + vector versus *atg27Δ / atg32Δ* + Bcl2-L-13 p = 0.0048, *atg32Δ* + Bcl2-L-13 versus *atg29Δ / atg32Δ* + vector p < 0.0001, *atg32Δ* + Bcl2-L-13 versus *atg29Δ / atg32Δ* + Bcl2-L-13 p < 0.0001, *atg29Δ / atg32Δ* + vector versus *atg29Δ / atg32Δ* + Bcl2-L-13 p = 0.9634, *atg32Δ* + Bcl2-L-13 versus *atg31Δ / atg32Δ* + vector p < 0.0001, *atg32Δ* + Bcl2-L-13 versus *atg31Δ / atg32Δ* + Bcl2-L-13 p < 0.0001, *atg31Δ / atg32Δ* + vector versus *atg31Δ / atg32Δ* + Bcl2-L-13 p = 0.7335, *atg32Δ* + Bcl2-L-13 versus *atg33Δ / atg32Δ* + vector p = 0.0074, *atg32Δ* + Bcl2-L-13 versus *atg33Δ / atg32Δ* + Bcl2-L-13 p = 0.0942, *atg33Δ / atg32Δ* + vector versus *atg33Δ / atg32Δ* + Bcl2-L-13 p = 0.0008.

A one-way ANOVA followed by Tukey–Kramer’s post hoc test.

In Table 1, phenotypes are indicated with plus and minus signs as follows: ++, a similar to or higher level than that in *atg32Δ*; +, a significantly higher level than the corresponding empty vector-transfected strain and lower level than *atg32Δ*; -, a similar level to the corresponding vector-transfected strain.

Table 1, Figure S2G.

For all groups, n = 3. *atg32Δ* + Bcl2-L-13 versus *atg1Δ* + vector p < 0.0001, *atg32Δ* + Bcl2-L-13 versus *atg1Δ* + Bcl2-L-13 p < 0.0001, *atg1Δ* + vector versus *atg1Δ* + Bcl2-L-13 p = 0.3443, *atg32Δ* + Bcl2-L-13 versus *atg2Δ* + vector p < 0.0001, *atg32Δ* + Bcl2-L-13 versus *atg2Δ* + Bcl2-L-13 p < 0.0001, *atg2Δ* + vector versus *atg2Δ* + Bcl2-L-13 p > 0.9999, *atg32Δ* + Bcl2-L-13 versus *atg3Δ / atg32Δ* + vector p < 0.0001, *atg32Δ* + Bcl2-L-13 versus *atg3Δ / atg32Δ* + Bcl2-L-13 p < 0.0001, *atg3Δ / atg32Δ* + vector versus *atg3Δ / atg32Δ* + Bcl2-L-13 p = 0.5470, *atg32Δ* + Bcl2-L-13 versus *atg4Δ / atg32Δ* + vector p < 0.0001, *atg32Δ* + Bcl2-L-13 versus *atg4Δ / atg32Δ* + Bcl2-L-13 p < 0.0001, *atg4Δ / atg32Δ* + vector versus *atg4Δ / atg32Δ* + Bcl2-L-13 p = 0.9891, *atg32Δ* + Bcl2-L-13 versus *atg5Δ / atg32Δ* + vector p < 0.0001, *atg32Δ* + Bcl2-L-13 versus *atg5Δ / atg32Δ* + Bcl2-L-13 p < 0.0001, *atg5Δ / atg32Δ* + vector versus *atg5Δ / atg32Δ* + Bcl2-L-13 p = 0.9981, *atg32Δ* + Bcl2-L-13 versus *atg6Δ / atg32Δ* + vector p < 0.0001, *atg32Δ* + Bcl2-L-13 versus *atg6Δ / atg32Δ* + Bcl2-L-13 p < 0.0001, *atg6Δ / atg32Δ* + vector versus *atg6Δ / atg32Δ* + Bcl2-L-13 p = 0.8586, *atg32Δ* + Bcl2-L-13 versus *atg7Δ* + vector p < 0.0001, *atg32Δ* + Bcl2-L-13 versus *atg7Δ* + Bcl2-L-13 p < 0.0001, *atg7Δ* + vector versus *atg7Δ* + Bcl2-L-13 p = 0.2844, *atg32Δ* + Bcl2-L-13 versus *atg8Δ / atg32Δ* + vector p < 0.0001, *atg32Δ* + Bcl2-L-13 versus *atg8Δ / atg32Δ* + Bcl2-L-13 p < 0.0001, *atg8Δ / atg32Δ* + vector versus *atg8Δ / atg32Δ* + Bcl2-L-13 p = 0.1185, *atg32Δ* + Bcl2-L-13 versus *atg9Δ* + vector p < 0.0001, *atg32Δ* + Bcl2-L-13 versus *atg9Δ* + Bcl2-L-13 p < 0.0001, *atg9Δ* + vector versus *atg9Δ* + Bcl2-L-13 p = 0.8550, *atg32Δ* + Bcl2-L-13 versus *atg10Δ / atg32Δ* + vector p < 0.0001, *atg32Δ* + Bcl2-L-13 versus *atg10Δ / atg32Δ* + Bcl2-L-13 p < 0.0001, *atg10Δ / atg32Δ* + vector versus *atg10Δ / atg32Δ* + Bcl2-L-13 p = 0.9726, *atg32Δ* + Bcl2-L-13 versus *atg11Δ / atg32Δ* + vector p = 0.0049, *atg32Δ* + Bcl2-L-13 versus *atg11Δ / atg32Δ* + Bcl2-L-13 p = 0.9867, *atg11Δ / atg32Δ* + vector versus *atg11Δ / atg32Δ* + Bcl2-L-13 p = 0.0057, *atg32Δ* + Bcl2-L-13 versus *atg12Δ / atg32Δ* + vector p < 0.0001, *atg32Δ* + Bcl2-L-13 versus *atg12Δ / atg32Δ* + Bcl2-L-13 p < 0.0001, *atg12Δ / atg32Δ* + vector versus *atg12Δ / atg32Δ* + Bcl2-L-13 p = 0.9715, *atg32Δ* + Bcl2-L-13 versus *atg13Δ / atg32Δ* + vector p < 0.0001, *atg32Δ* + Bcl2-L-13 versus *atg13Δ / atg32Δ* + Bcl2-L-13 p < 0.0001, *atg13Δ / atg32Δ* + vector versus *atg13Δ / atg32Δ* + Bcl2-L-13 p = 0.2283, *atg32Δ* + Bcl2-L-13 versus *atg14Δ* + vector p < 0.0001, *atg32Δ* + Bcl2-L-13 versus *atg14Δ* + Bcl2-L-13 p < 0.0001, *atg14Δ* + vector versus *atg14Δ* + Bcl2-L-13 p = 0.9833, *atg32Δ* + Bcl2-L-13 versus *atg16Δ / atg32Δ* + vector p < 0.0001, *atg32Δ* + Bcl2-L-13 versus *atg16Δ / atg32Δ* + Bcl2-L-13 p < 0.0001, *atg16Δ / atg32Δ* + vector versus *atg16Δ / atg32Δ* + Bcl2-L-13 p = 0.3889, *atg32Δ* + Bcl2-L-13 versus *atg17Δ / atg32Δ* + vector p < 0.0001, *atg32Δ* + Bcl2-L-13 versus *atg17Δ / atg32Δ* + Bcl2-L-13 p < 0.0001, *atg17Δ / atg32Δ* + vector versus *atg17Δ / atg32Δ* + Bcl2-L-13 p = 0.8964, *atg32Δ* + Bcl2-L-13 versus *atg18Δ / atg32Δ* + vector p < 0.0001, *atg32Δ* + Bcl2-L-13 versus *atg18Δ / atg32Δ* + Bcl2-L-13 p < 0.0001, *atg18Δ / atg32Δ* + vector versus *atg18Δ / atg32Δ* + Bcl2-L-13 p = 0.1051, *atg32Δ* + Bcl2-L-13 versus *atg19Δ / atg32Δ* + vector p < 0.0001, *atg32Δ* + Bcl2-L-13 versus *atg19Δ / atg32Δ* + Bcl2-L-13 p = 0.4043, *atg19Δ / atg32Δ* + vector versus *atg19Δ / atg32Δ* + Bcl2-L-13 p < 0.0001, *atg32Δ* + Bcl2-L-13 versus *atg20Δ / atg32Δ* + vector p < 0.0001, *atg32Δ* + Bcl2-L-13 versus *atg20Δ / atg32Δ* + Bcl2-L-13 p < 0.0001, *atg20Δ / atg32Δ* + vector versus *atg20Δ / atg32Δ* + Bcl2-L-13 p < 0.0001, *atg32Δ* + Bcl2-L-13 versus *atg21Δ / atg32Δ* + vector p < 0.0001, *atg32Δ* + Bcl2-L-13 versus *atg21Δ / atg32Δ* + Bcl2-L-13 p < 0.0001, *atg21Δ / atg32Δ* + vector versus *atg21Δ / atg32Δ* + Bcl2-L-13 p = 0.0099, *atg32Δ* + Bcl2-L-13 versus *atg23Δ / atg32Δ* + vector p < 0.0001, *atg32Δ* + Bcl2-L-13 versus *atg23Δ / atg32Δ* + Bcl2-L-13 p = 0.0062, *atg23Δ / atg32Δ* + vector versus *atg23Δ / atg32Δ* + Bcl2-L-13 p = 0.0003, *atg32Δ* + Bcl2-L-13 versus *atg24Δ / atg32Δ* + vector p < 0.0001, *atg32Δ* + Bcl2-L-13 versus *atg24Δ / atg32Δ* + Bcl2-L-13 p < 0.0001, *atg24Δ / atg32Δ* + vector versus *atg24Δ / atg32Δ* + Bcl2-L-13 p < 0.0001, *atg32Δ* + Bcl2-L-13 versus *atg27Δ / atg32Δ* + vector p = 0.0002, *atg32Δ* + Bcl2-L-13 versus *atg27Δ / atg32Δ* + Bcl2-L-13 p = 0.7620, *atg27Δ / atg32Δ* + vector versus *atg27Δ / atg32Δ* + Bcl2-L-13 p = 0.0004, *atg32Δ* + Bcl2-L-13 versus *atg29Δ / atg32Δ* + vector p < 0.0001, *atg32Δ* + Bcl2-L-13 versus *atg29Δ / atg32Δ* + Bcl2-L-13 p < 0.0001, *atg29Δ / atg32Δ* + vector versus *atg29Δ / atg32Δ* + Bcl2-L-13 p = 0.4606, *atg32Δ* + Bcl2-L-13 versus *atg31Δ / atg32Δ* + vector p < 0.0001, *atg32Δ* + Bcl2-L-13 versus *atg31Δ / atg32Δ* + Bcl2-L-13 p < 0.0001, *atg31Δ / atg32Δ* + vector versus *atg31Δ / atg32Δ* + Bcl2-L-13 p = 0.1497, *atg32Δ* + Bcl2-L-13 versus *atg33Δ / atg32Δ* + vector p < 0.0001, *atg32Δ* + Bcl2-L-13 versus *atg33Δ / atg32Δ* + Bcl2-L-13 p = 0.1576, *atg33Δ / atg32Δ* + vector versus *atg33Δ / atg32Δ* + Bcl2-L-13 p < 0.0001.

A one-way ANOVA followed by Tukey–Kramer’s post hoc test.

In Table 1, phenotypes are indicated with plus and minus signs as follows: ++, a similar to or higher level than that in *atg32Δ*; +, a significantly higher level than the corresponding empty vector-transfected strain and lower level than *atg32Δ*; -, a similar level to the corresponding vector-transfected strain.

Figure S3E.

For all groups, n = 3. vector versus FLAG-ULK1 wild-type p = 0.0002, vector versus FLAG-ULK1 K46N p = 0.2912, vector versus FLAG-ULK1 F357A V360A p = 0.1024, FLAG-ULK1 wild-type versus FLAG-ULK1 K46N p = 0.0013, FLAG-ULK1 wild-type versus FLAG-ULK1 F357A V360A p = 0.0032, FLAG-ULK1 K46N versus FLAG-ULK1 F357A V360A p = 0.8654.

A one-way ANOVA followed by Tukey–Kramer’s post hoc test.

Figure S4B.

For all groups, n = 3. siCtrl versus siULK1 p < 0.0001, siCtrl versus siFIP200 p < 0.0001, siCtrl versus siATG13 p < 0.0001, siCtrl versus siATG101 p < 0.0001.

An unpaired, two-tailed t test.

Figure S4D.

For all groups, n = 3. siCtrl versus siULK1 p < 0.0001, siCtrl versus siFIP200 p < 0.0001, siCtrl versus siATG13 p < 0.0001, siCtrl versus siATG101 p < 0.0001, siULK1 versus siFIP200 p = 0.0051, siULK1 versus siATG13 p = 0.9994, siULK1 versus siATG101 p = 0.0021, siFIP200 versus siATG13 p = 0.0037, siFIP200 versus siATG101 p = 0.9656, siATG13 versus siATG101 p = 0.0015.

A one-way ANOVA followed by Tukey–Kramer’s post hoc test.

## Supplemental Information

Document S1. Figures S1–S4 and Tables S1–S3

Document S2. Article plus Supplemental Information
