# Supplementary material for: A Mammalian Mitophagy Receptor, Bcl2-L-13, Recruits the ULK1 Complex to Induce Mitophagy
Source: Cell Rep. 2019 Jan 8;26(2):338–345.e6. doi: 10.1016/j.celrep.2018.12.050 (PMC6326162; doi:10.1016/j.celrep.2018.12.050)
Supplement: Document S1. Figures S1–S4 and Tables S1–S3 [file mmc1.pdf]

Cell Reports, Volume *26*

Supplemental Information

A Mammalian Mitophagy Receptor,

Bcl2-L-13, Recruits the ULK1 Complex

to Induce Mitophagy

Tomokazu Murakawa, Koji Okamoto, Shigemiki Omiya, Manabu Taneike, Osamu Yamaguchi, and Kinya Otsu

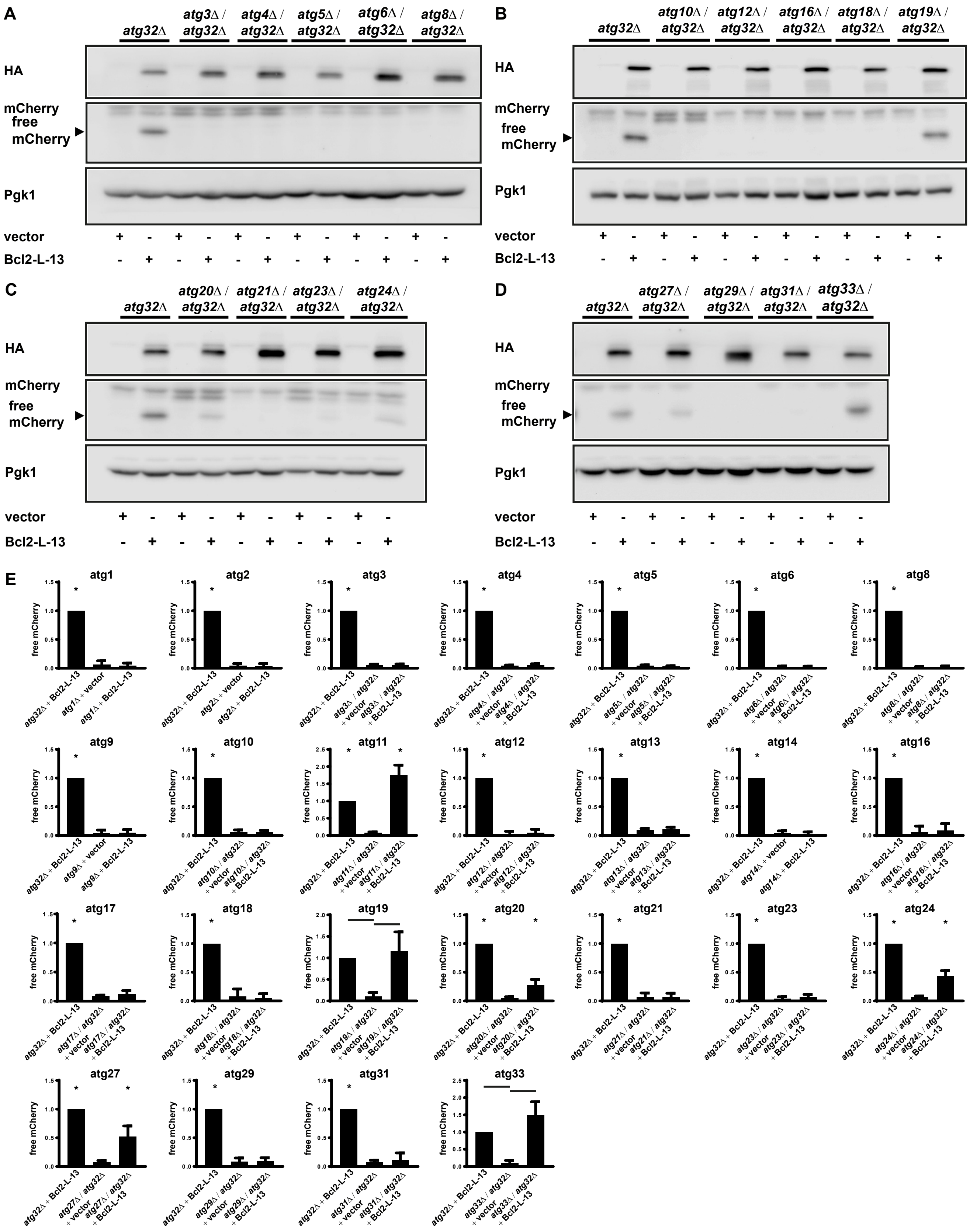

Figure S1. Results of Mitophagy Assay in Glycerol Medium, Related to Figure 1 and Table 1.
(A-E) Yeast cells transfected with HA-Bcl2-L-13 or empty vector were collected 72 h after induction of mitophagy in SD-G medium and subjected to western blotting for mCherry. Generation of free mCherry is indicated by an arrow-head. Yeast strains are *atg32*Δ, *atg3*Δ/*atg32*Δ, *atg4*Δ/*atg32*Δ, *atg5*Δ/*atg32*Δ, *atg6*Δ/*atg32*Δ or *atg8*Δ/*atg32*Δ in (A), *atg32*Δ, *atg10*Δ/*atg32*Δ, *atg12*Δ/*atg32*Δ, *atg16*Δ/*atg32*Δ, *atg18*Δ/*atg32*Δ or *atg19*Δ/*atg32*Δ in (B), *atg32*Δ, *atg20*Δ/*atg32*Δ, *atg21*Δ/*atg32*Δ, *atg23*Δ/*atg32*Δ or *atg24*Δ/*atg32*Δ in (C), *atg32*Δ, *atg27*Δ/*atg32*Δ, *atg29*Δ/*atg32*Δ, *atg31*Δ/*atg32*Δ or *atg33*Δ/*atg32*Δ in (D). Quantitative analysis for free mCherry in (A-D) is shown in (E). The value for *atg32*Δ cells transfected with HA-Bcl2-L-13 in each experiment was set equal to 1 (n = 3). *P < 0.05 versus all other groups. Bars in graphs indicate P < 0.05. Results are shown as mean ± SEM.

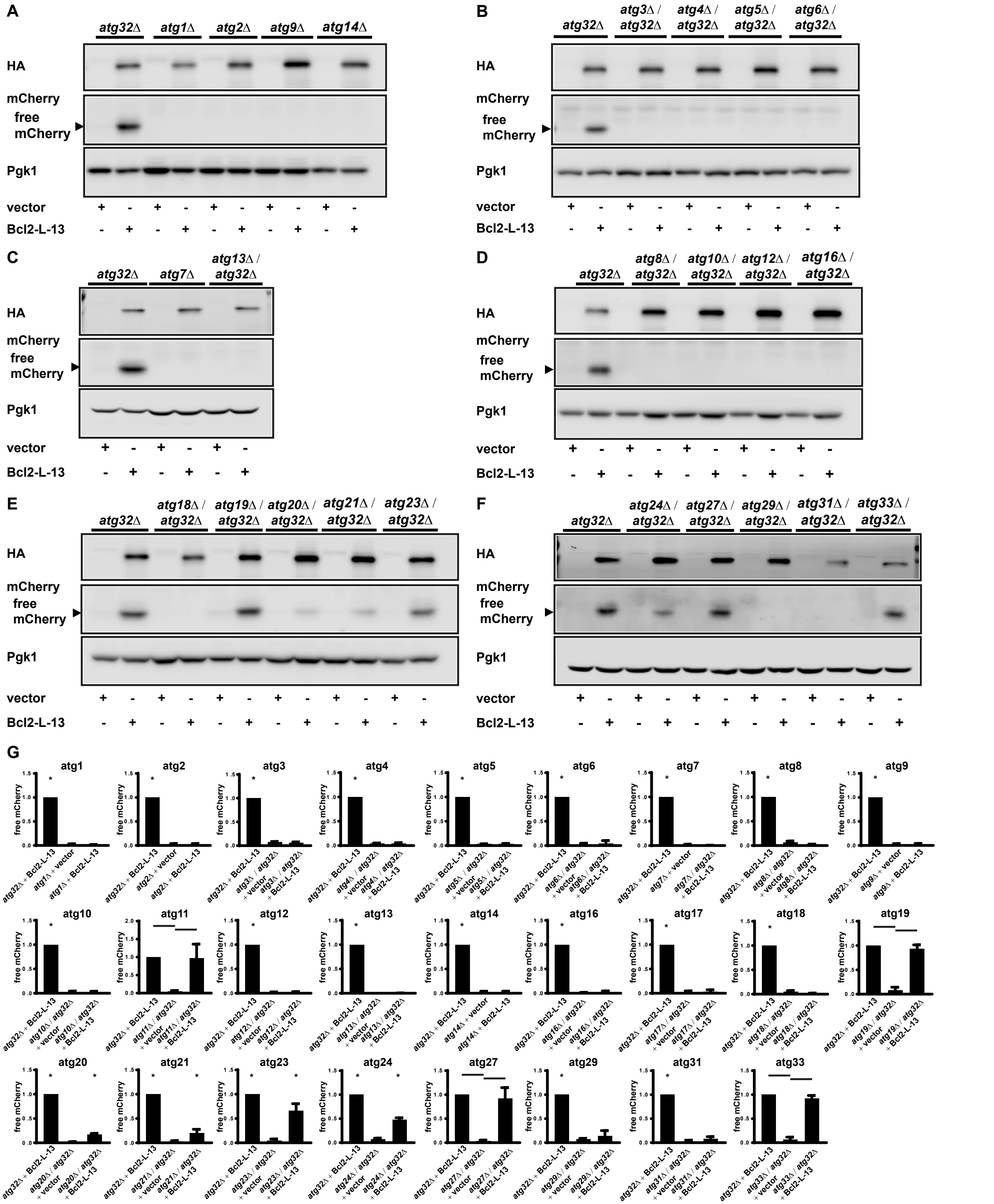

Figure S2. Results of Mitophagy Assay in Nitrogen-Starvation Medium, Related to Figure 1.

(A-G) Yeast cells transfected with HA-Bcl2-L-13 or empty vector were collected 48 h after induction of mitophagy in SD-N medium and subjected to western blotting for mCherry. Generation of free mCherry is indicated by an arrow-head. Yeast strains are *atg32*Δ, *atg1*Δ, *atg2*Δ, *atg9*Δ or *atg14*Δ in (A), *atg32*Δ, *atg3*Δ/*atg32*Δ, *atg4*Δ/*atg32*Δ, *atg5*Δ/*atg32*Δ or *atg6*Δ/*atg32*Δ in (B), *atg32*Δ, *atg7*Δ or *atg13*Δ/*atg32*Δ in (C), *atg32*Δ, *atg8*Δ/*atg32*Δ, *atg10*Δ/*atg32*Δ, *atg12*Δ/*atg32*Δ or *atg16*Δ/*atg32*Δ in (D), *atg32*Δ, *atg18*Δ/*atg32*Δ, *atg19*Δ/*atg32*Δ, *atg20*Δ/*atg32*Δ, *atg21*Δ/*atg32*Δ or *atg23*Δ/*atg32*Δ in (E), *atg32*Δ, *atg24*Δ/*atg32*Δ, *atg27*Δ/*atg32*Δ, *atg29*Δ/*atg32*Δ, *atg31*Δ/*atg32*Δ or *atg33*Δ/*atg32*Δ in (F). Quantitative analysis for free mCherry in (A-F) is shown in (G). The value from *atg32*Δ cells transfected with HA-Bcl2-L-13 in each experiment was set equal to 1 (n = 3). *P < 0.05 versus all other groups. Bars in graphs indicate P < 0.05. Results are shown as mean ± SEM.

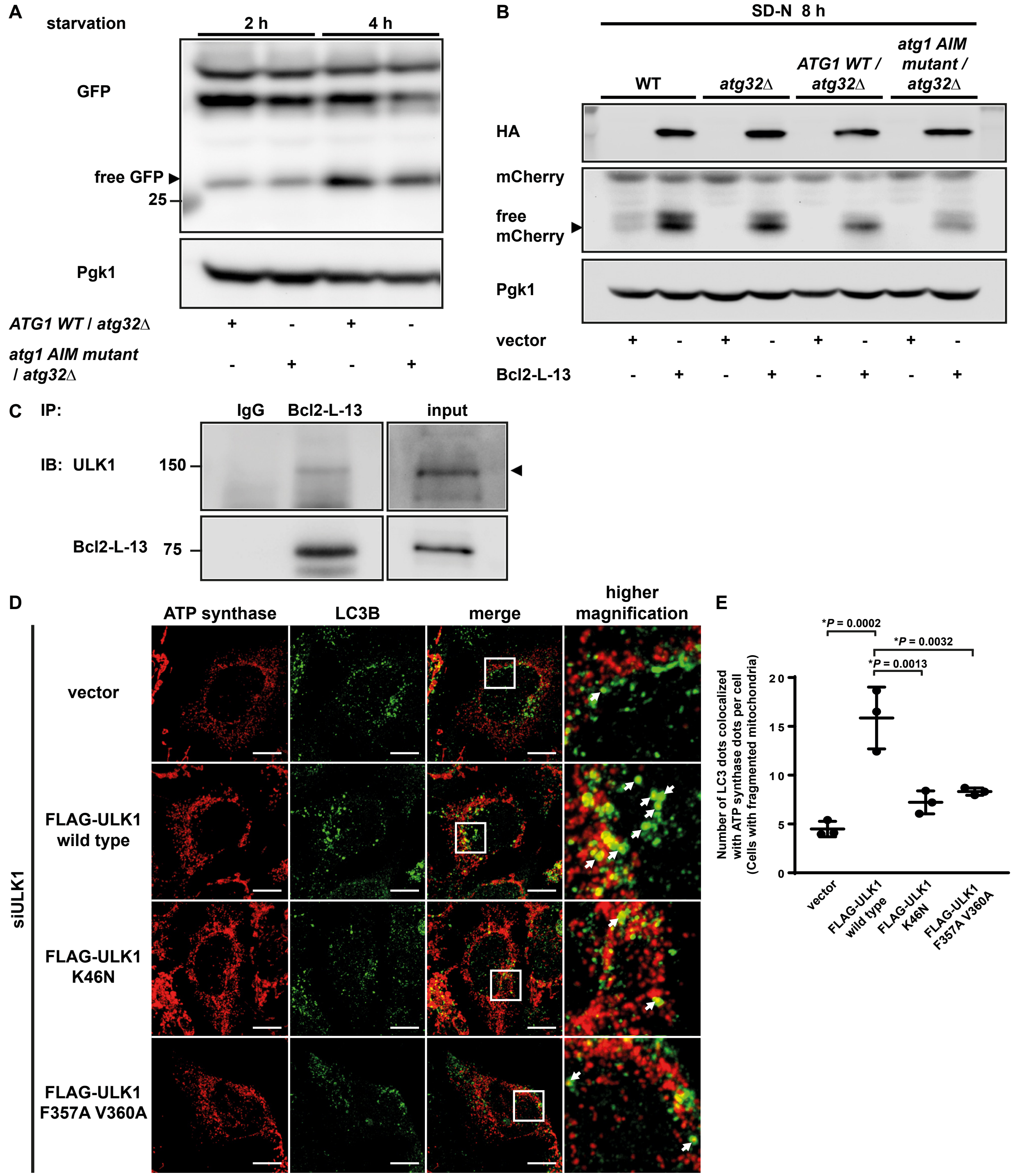

Figure S3. ULK1–LC3B (Atg1–Atg8) Interaction through the LIR (AIM) Motif Is Important for Bcl2-L-13-mediated Mitophagy, Related to Figure 2.

(A) *ATG1 WT*/*atg32*Δ and *atg1 AIM mutant*/*atg32*Δ cells expressing GFP-Atg8 were grown to mid-log phase in rich dextrose medium, incubated for the indicated time points in starvation medium (SD-N), and subjected to western blotting. Generation of free GFP (indicated by an arrow-head) indicates transport of autophagosomes to the vacuole. (B) *ATG1 WT*/*atg32*Δ and *atg1 AIM mutant KI*/*atg32*Δ cells transfected with HA-Bcl2-L-13 or empty vector were collected 8 h after induction of mitophagy in SD-N medium and subjected to western blotting for mCherry. (C) Endogenous interaction between Bcl2-L-13 and ULK1. HEK292A cells were treated with 15 μM CCCP for 6 h. One hundred nM bafilomycin A1 was added 3 h before sampling. Then, cells were lysed and immunoprecipitated with anti-Bcl2-L-13 antibody. Co-precipitated ULK1 was detected by immunoblotting. ULK1 is indicated by an arrow-head. (D and E) Colocalization of autophagosomes and mitochondria in HEK293A cells. HEK293A cells were transfected with ULK1-specific siRNA for 72 h followed by transfection of HA-Bcl2-L-13 and FLAG tagged wild-type ULK1 or ULK1 mutants. Forty-two h after transfection, cells were treated with 100 nM bafilomycin A1 for 6 h and immunostained with anti-LC3B and anti-ATP synthase antibodies. Images in the box at higher magnification are shown in the right panels. White arrows indicate colocalization of LC3B and ATP synthase double positive dots. The number of LC3B dots colocalized with ATP synthase dots per cell is shown in (E). At least 20 cells were counted for each group (n = 3). Scale bar: 10 μm. *$P < 0.05$. Results are shown as mean ± SEM.

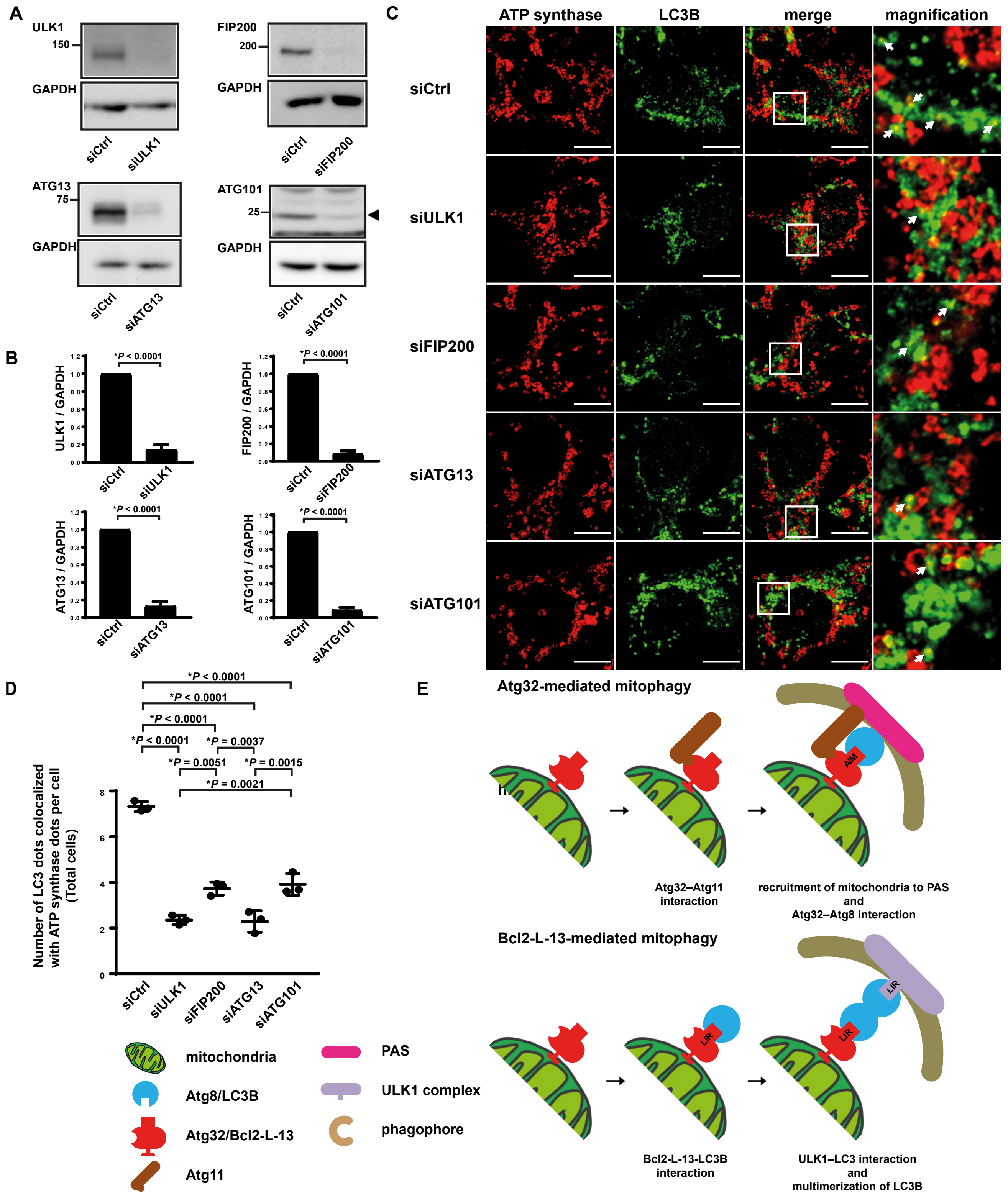

Figure S4. The ULK1 Complex Is Important for Bcl2-L-13-mediated Mitophagy, Related to Figure 3.
(A and B) HEK293A cells were transfected with indicated siRNAs and analyzed by immunoblotting. Densitometric analysis isshown in (B). The value for cells treated with siCtrl in each experiment was set equal to 1 (n = 3). *P < 0.05 versus control. Quantitative results are shown as mean ± SEM. (C and D) Colocalization of autophagosomes and mitochondria in HEK293A cells. HEK293A cells were transfected with indicated siRNA for 72 h followed by 15 μM CCCP treatment for 6 h. One hundred nM bafilomycin A1 was added 3 h before fixation. Cells were immunostained with anti-LC3B and anti-ATP synthase antibodies. Images in the box at higher magnification are shown in the right panels. White arrows indicate colocalization of LC3B and ATP synthase double positive dots. The number of LC3B dots colocalized with ATP synthase dots per cell is shown in (D). At least 20 cells were counted for each group (n = 3). Scale bar: 10 μm. *P < 0.05. Results are shown as mean ± SEM. (E) Conceivable Models of Atg32-mediated and Bcl2-L-13-mediated Mitophagy. Atg32-mediated mitophagy: When mitophagy is induced, Atg11 interacts with Atg32 in yeast. The Atg11–Atg32 complex recruits mitochondria to the PAS. Mitochondria are surrounded by the phagophore through an interaction between Atg32 and Atg8 via AIM. Bcl2-L-13-mediated mitophagy: Upon mitophagy induction, LC3B is recruited to Bcl2-L-13, while the assembly of the ULK1 complex and the interaction between ULK1 and LC3B through the LIR motif take place. How Bcl2-L-13 interacts with the ULK1 complex is unclear, but it is possible that they interact though LC3B multimerization.

Table S1. Comparison between *ATG* Gene Requirements for Bcl2-L-13-mediated Mitophagy and for Canonical Yeast Mitophagy, Related to Figure 1 and Table 1.

genotype	yeast mitophagy	Bcl2-L-13-mediated mitophagy in yeast
atg1Δ	-	-
atg2Δ	-	-
atg3Δ	-	-
atg4Δ	-	-
atg5Δ	-	-
atg6Δ	-	-
atg7Δ	-	-
atg8Δ	-	-
atg9Δ	-	-
atg10Δ	-	-
atg11Δ	-	++
atg12Δ	-	-
atg13Δ	++	-
atg14Δ	-	-
atg16Δ	-	-
atg17Δ	++	-
atg18Δ	-	-
atg19Δ	+++	++
atg20Δ	+	+
atg21Δ	+++	-
atg23Δ	++	-
atg24Δ	+	+
atg27Δ	++	+
atg29Δ	+++	-
atg31Δ	+++	-
atg32Δ	-	++

Bcl2-L-13-mediated mitophagy was assayed by protein degradation assay using mitochondria targeted mCherry-DHFR expressing cells grown in glycerol medium for 72 h. The requirement of certain *ATG* genes for the canonical yeast mitophagy has previously been described (Okamoto et al., 2009). Phenotypes in Bcl2-L-12-mediated mitophagy are indicated with plus and minus signs as follows: ++, a similar to or higher level than that in *atg32Δ;* +, a significantly higher level than the corresponding empty vector-transfected strain and lower level than *atg32Δ*; -, a similar level to the corresponding vector-transfected strain. Phenotypes in canonical yeast mitophagy are indicated with plus and minus signs as follows: +++, wild-type-like; ++, slightly defective; +, partially defective; -, completely defective.

Table S2. Yeast Strains Used in This Study, Related to STAR Methods

Name	Genotype
KOY1387	*BY4741 his3Δ1 leu2Δ0 met15Δ0 ura3Δ0 TEFp-mito-DHFR-mcherry::CgHIS3*
KOY1422	*BY4741 his3Δ1 leu2Δ0 met15Δ0 ura3Δ0 TEFp-mito-DHFR mcherry::CgHIS3 atg32::kanMX6*
KOY1424	*BY4741 his3Δ1 leu2Δ0 met15Δ0 ura3Δ0 TEFp-mito-DHFR-mcherry::CgHIS3 atg7::kanMX6*
KOY1569	*BY4741 his3Δ1 leu2Δ0 met15Δ0 ura3Δ0 TEFp-mito-DHFR-mcherry::CgHIS3 atg1::kanMX6*
KOY1539	*BY4741 his3Δ1 leu2Δ0 met15Δ0 ura3Δ0 TEFp-mito-DHFR-mcherry::CgHIS3 atg2::kanMX6*
KOY1545	*BY4741 his3Δ1 leu2Δ0 met15Δ0 ura3Δ0 TEFp-mito-DHFR-mcherry::CgHIS3 atg9::kanMX6*
KOY1547	*BY4741 his3Δ1 leu2Δ0 met15Δ0 ura3Δ0 TEFp-mito-DHFR-mcherry::CgHIS3 atg14::kanMX6*
YMT7	*BY4741 his3Δ1 leu2Δ0 met15Δ0 ura3Δ0 TEFp-mito-DHFR-mcherry::CgHIS3 atg11::natNT2 atg32::kanMX6*
YMT9	*BY4741 his3Δ1 leu2Δ0 met15Δ0 ura3Δ0 TEFp-mito-DHFR-mcherry::CgHIS3 atg13::natNT2 atg32::kanMX6*
YMT11	*BY4741 his3Δ1 leu2Δ0 met15Δ0 ura3Δ0 TEFp-mito-DHFR-mcherry::CgHIS3 atg17::natNT2 atg32::kanMX6*
YMT13	*BY4741 his3Δ1 leu2Δ0 met15Δ0 ura3Δ0 TEFp-mito-DHFR-mcherry::CgHIS3 atg24::natNT2 atg32::kanMX6*
YMT55	*BY4741 his3Δ1 leu2Δ0 met15Δ0 ura3Δ0 TEFp-mito-DHFR-mcherry::CgHIS3 atg3::natNT2 atg32::kanMX6*
YMT57	*BY4741 his3Δ1 leu2Δ0 met15Δ0 ura3Δ0 TEFp-mito-DHFR-mcherry::CgHIS3 atg4::natNT2 atg32::kanMX6*
YMT59	*BY4741 his3Δ1 leu2Δ0 met15Δ0 ura3Δ0 TEFp-mito-DHFR-mcherry::CgHIS3 atg5::natNT2 atg32::kanMX6*
YMT39	*BY4741 his3Δ1 leu2Δ0 met15Δ0 ura3Δ0 TEFp-mito-DHFR-mcherry::CgHIS3 atg6::natNT2 atg32::kanMX6*
YMT61	*BY4741 his3Δ1 leu2Δ0 met15Δ0 ura3Δ0 TEFp-mito-DHFR-mcherry::CgHIS3 atg8::natNT2 atg32::kanMX6*
YMT69	*BY4741 his3Δ1 leu2Δ0 met15Δ0 ura3Δ0 TEFp-mito-DHFR-mcherry::CgHIS3 atg10::natNT2 atg32::kanMX6*
YMT25	*BY4741 his3Δ1 leu2Δ0 met15Δ0 ura3Δ0 TEFp-mito-DHFR-mcherry::CgHIS3 atg12::natNT2 atg32::kanMX6*
YMT27	*BY4741 his3Δ1 leu2Δ0 met15Δ0 ura3Δ0 TEFp-mito-DHFR-mcherry::CgHIS3 atg16::natNT2 atg32::kanMX6*
YMT29	*BY4741 his3Δ1 leu2Δ0 met15Δ0 ura3Δ0 TEFp-mito-DHFR-mcherry::CgHIS3 atg18::natNT2 atg32::kanMX6*
YMT67	*BY4741 his3Δ1 leu2Δ0 met15Δ0 ura3Δ0 TEFp-mito-DHFR-mcherry::CgHIS3 atg19::natNT2 atg32::kanMX6*
YMT47	*BY4741 his3Δ1 leu2Δ0 met15Δ0 ura3Δ0 TEFp-mito-DHFR-mcherry::CgHIS3 atg20::natNT2 atg32::kanMX6*
YMT51	*BY4741 his3Δ1 leu2Δ0 met15Δ0 ura3Δ0 TEFp-mito-DHFR-mcherry::CgHIS3 atg23::natNT2 atg32::kanMX6*
YMT65	*BY4741 his3Δ1 leu2Δ0 met15Δ0 ura3Δ0 TEFp-mito-DHFR-mcherry::CgHIS3 atg27::natNT2 atg32::kanMX6*
YMT35	*BY4741 his3Δ1 leu2Δ0 met15Δ0 ura3Δ0 TEFp-mito-DHFR-mcherry::CgHIS3 atg29::natNT2 atg32::kanMX6*
YMT37	*BY4741 his3Δ1 leu2Δ0 met15Δ0 ura3Δ0 TEFp-mito-DHFR-mcherry::CgHIS3 atg31::natNT2 atg32::kanMX6*
YMT77	*BY4741 his3Δ1 leu2Δ0 met15Δ0 ura3Δ0 TEFp-mito-DHFR-mcherry::CgHIS3 atg33::natNT2 atg32::kanMX6*
YMT71	*BY4741 his3Δ1 leu2Δ0 met15Δ0 ura3Δ0 TEFp-mito-DHFR-mcherry::CgHIS3 atg1::ATG1::kanMX4*
YMT73	*BY4741 his3Δ1 leu2Δ0 met15Δ0 ura3Δ0 TEFp-mito-DHFR-mcherry::CgHIS3 atg1::ATG1 AIM mut::kanMX4*
YMT79	*BY4741 his3Δ1 leu2Δ0 met15Δ0 ura3Δ0 TEFp-mito-DHFR-mcherry::CgHIS3 atg1::ATG1::kanMX4 atg32::natNT2*
YMT81	*BY4741 his3Δ1 leu2Δ0 met15Δ0 ura3Δ0 TEFp-mito-DHFR-mcherry::CgHIS3 atg1::ATG1 AIM mut::kanMX4 atg32::natNT2*

Table S3. Primers Used in This Study, Related to STAR Methods

Name	5´-3´ oligonucleotide
ATG3 cassette - Fw	TATCAAGCTAGCTAGAAGTTAGGAACAAAGAAGTACAAAGGAGTAAATAC<br>AATTTTATTATCCGTACGCTGCAGGTCGAC
ATG3 cassette - Rv	TCTTGTTTATCCTGTTTTTTGACCACCTGGCTTGCAGCTAATAGTGAAAAA<br>ACACAAATTTATCGATGAATTCGAGCTCG
ATG4 cassette - Fw	CACACAATAACGTTAGTAGATGAAGAATGGACGACTTCTTATCACGTATAG<br>GAGTGATATACCGTACGCTGCAGGTCGAC
ATG4 cassette - Rv	CTCAGTTTTAGGGAATATATTAAAACAAGTATATATGCTTATGAACTAGTGA<br>ATTCCTTACAATCGATGAATTCGAGCTC
ATG5 cassette - Fw	GCGAATGAATTTTTGTTCTTTTGGTTCTAGAAGAACGGAGATAGGAAACCT<br>ATGATGTAAGTCGTACGCTGCAGGTCGAC
ATG5 cassette - Rv	AACGTCCAAGTTATTTTCTGCGATATTTGAATGACACTTTTAAATGCGTATA<br>TAACAGCTCATCGATGAATTCGAGCTCG
ATG6 cassette - Fw	AGTTCAGTTACGTTTTATGGCAGTCACTGTTTTCGCAAAGACTCCCAGACA<br>CGGGCATTAAACGTACGCTGCAGGTCGAC
ATG6 cassette - Rv	AAGTGGAAAAAGAAATTTTCCCTTTATCACATTTATGAAAAAATGCATTTAT<br>ATGAACTACATCGATGAATTCGAGCTCG
ATG8 cassette - Fw	TGATAAGAGAATCTAATAATTGTAAAGTTGAGAAAATCATAATAAAAATAAT<br>TACTAGAGACCGTACGCTGCAGGTCGAC
ATG8 cassette - Rv	CCTATAATTTCGATTTTAGATGTTAACGCTTCATTTCTTTTCATATAAAAGA<br>CTACCTGCCATCGATGAATTCGAGCTCG
ATG10 cassette - Fw	ATGAGAAGAGAACACCATGAAAAAAAAAAAAAAAGGGCTAAAAAACAGAA<br>TTATCAGACTTGCGTACGCTGCAGGTCGAC
ATG10 cassette - Rv	ATATATATATATATTTACATAGATGATTGCATAGTGTTTTAAAAAGCTTTCCT<br>AGGTTAAGATCGATGAATTCGAGCTCG
ATG11-flk[942-1003]-S1FW	ATTATTTTAGTGTACTGTTGTTGTTCGGAAAGTACTTCTTTTATTTTCTTTTA<br>TACATCATGCGTACGCTGCAGGTCGAC
ATG11-flk[4592-4532]-S2RV	GTTAAATAGATACATAATTAAAATCTTGTCATTTGTGACAAACGTTTAGCAC<br>TGTTCAAACATCGATGAATTCGAGCTCG
ATG12 cassette - Fw	TGACGAAAGCTCCATCATATCCACAAATAATGGAACGGCAATGGAAAGAT<br>CCAGAAATAATCCGTACGCTGCAGGTCGAC
ATG12 cassette - Rv	TCGTACGGGATTTTTGATCGACTGTAGGTTTTCTTCTTAGACCATTCCAGC<br>GCCCGGGTATATCGATGAATTCGAGCTC
ATG13-flk[939-1000]-S1FW	CCTTCCAGGCTCAAGTCTTGAAAAGAAAGCAGAACATACAGCCCGGTTGA<br>ATAGCATGAGTCCGTACGCTGCAGGTCGAC
ATG13-flk[3264-3204]-S2RV	GATTATTTTTCTTTAGTTGTGCCCTTTAAAATAAAACTTTACCATTTTTAACC<br>TTCTTTAGATCGATGAATTCGAGCTCG
ATG16 cassette - Fw	CAAGGAGGCCCATTTAAATGAGTTGTTTCAAGATAATAGTGGCGCCATTG<br>GTGGCAACATTGCGTACGCTGCAGGTCGAC
ATG16 cassette - Rv	TGCACCACAATGATTTTATTTTCTTTTGTATGCATTTTGTGACGATTTGACA<br>ACTGATGCAATCGATGAATTCGAGCTCG
ATG17-flk[942-1003]-S1FW	GTCGCATAACGTACGATAAATTCGATACTGCGAGGATATTATCAACGTATT<br>TAACACCTATGCGTACGCTGCAGGTCGAC
ATG17-flk[2296-2236]-S2RV	CAATTATTGAATCTTTGTACCGTATCCTTTTTTTCCTTTTTTCTAAGGATTCT<br>TCACGTTGATCGATGAATTCGAGCTCG
ATG18 cassette - Fw	TTAATCAGTTAGTAATAGTGTTCCAGTTAACTCTGTATCCTTTTCTTCTTCG<br>GCCTGACACGTACGCTGCAGGTCGAC
ATG18 cassette - Rv	TATAAACTATATTGGTATGCGTTGTGACGTACGGAAGGCAGCGCGAGACA<br>CTTCCGTGAATCGATGAATTCGAGCTCG
ATG19 cassette - Fw	GAATCGAGGTAATTGCGGCGGCACTTGCTTCAGTAACGCCCAAAGGAGA<br>GTTCTGGTAAATGCGTACGCTGCAGGTCGAC
ATG19 cassette - Rv	GTATGTGAAAAGGTACTCATTGCTGTATAAAAATAGAGTTTGACCTAGAGT<br>TCTTCCCAAGATCGATGAATTCGAGCTCG

Table S3. Primers Used in This Study (continued)

Name	5´-3´ oligonucleotide
ATG20 cassette - Fw	CGAATACTGGTGGTGCAATTGAACATACCATCTAGAAAAGTTGTCTTCTAC AGCACACAATGCGTACGCTGCAGGTCGAC
ATG20 cassette - Rv	ACGTTGTTTATATATATATATATCAAGTATGCTATAACGCTAAAAAAAAATG CTCATAATAATCGATGAATTCGAGCTCG
ATG21 cassette - Fw	AATTTGACCAACAAAAGACAATTCCACTCCTTTGGATTTGAAATAGACAGA TAGAAAAGGATCGTACGCTGCAGGTCGAC
ATG21 cassette - Rv	TAGCAATTCACACGTGAATACGTACAATATCTATTAAGATTATGAAAACTG CACATATGCAATCGATGAATTCGAGCTCG
ATG23 cassette - Fw	TCTTGTTCCACATTTTAACCTCGTTGTTCTATAAGGTAACAAAATAAAGTGA AGAAGTAAATCGTACGCTGCAGGTCGAC
ATG23 cassette - Rv	GTTTACTACACTTTCAGAAATGCCAATCTTTGGTGGCGATTTGCTAGCAAC CAAGAATGAGATCGATGAATTCGAGCTCG
ATG24-flk[942-1003]-S1FW	TAAGCCGTTATTAAACTTTATTTACGGTATACCACAATACTGCTCTTTTTGT TGAGGATATGCGTACGCTGCAGGTCGAC
ATG24-flk[2333-2273]-S2RV	ATCAGATAATCGTAGTGCCCAAGGTATTATCAGTAGTAATGGGAAAACATT AAGAGCACCAATCGATGAATTCGAGCTCG
ATG27 cassette - Fw	TGTAGATTTATATCTGAGATCTTCAATCGATGCGATAGATAAAGGTAAGGA AAGCTTTCACGCGTACGCTGCAGGTCGAC
ATG27 cassette - Rv	GCCTAAGATTCTCGAAACCACTTCCTTGCAGAATTCAGGTAAGGAAGTGA GAAATTGAGTAATCGATGAATTCGAGCTCG
ATG29 cassette - Fw	ATACTTATCAAGAAGAGACATACATAATTGTACTGCTGTGCATTTTCCTACT TGACTTTCCGTACGCTGCAGGTCGAC
ATG29 cassette - Rv	AAACGCCGGGCCTTTTTGGCCGACAGTTGGTTTTTTGATTGTGCTTGTGA AAGATGTAAAATCGATGAATTCGAGCTCG
ATG31 cassette - Fw	TAAACGTCGCATTCCCATTTTCTTATTAAGCCGGTAAACATTGCTGAAATC TGCGAACAGGACGTACGCTGCAGGTCGAC
ATG31 cassette - Rv	AGAACATATACCTACATAAACAAGTTAAGAGAGTCTCATCCATGCGGCTTC ATTTTTGCTTATCGATGAATTCGAGCTCG
ATG32 cassette - Fw	TAAGCAATATTGAAGTCCTAATCACAAAAGCAAAAAAAATCTGCCAGGAAC AGTAAACATATGCGTACGCTGCAGGTCGAC
ATG32 cassette - Rv	ACAGAAGTGATAGTAAAAAAGTGAGTAGGAACGTGTATGTTTGTGTATATT GGAAAAAGGATCGATGAATTCGAGCTC
ATG1-integ-Fw	GAAGCTACCCCATATTTTCAAATCTCTTTTACAACACCAGACGAGAAATTA AGAAAATGGGAGACATTAAAAATAAAGAT
ATG1-integ-Rv	CTTGAAAATATAGCAGGTCATTTGTACTTAATAAGAAAACCATATTATGCAT CACTTAGAAAAACTCATCGAGCATCAAA
ULK1 K46N-Fw	GACCTGGAGGTGGCCGTCAACTGCATTAACAAGAAGAAC
ULK1 K46N-Rv	GTTCTTCTTGTTAATGCAGTTGACGGCCACCTCCAGGTC
ULK1F357A V360A-Fw	TGTGACACAGATGACGCTGTCATGGCCCCAGCCCAGTTTCCA
ULK1 F357A V360A-Rv	TGGAAACTGGGCTGGGGCCATGACAGCGTCATCTGTGTCACA
